# Light-Controlled Interconvertible Self-Assembly of Non-Photoresponsive Suprastructures

**DOI:** 10.3390/molecules29204842

**Published:** 2024-10-12

**Authors:** Wentao Yu, Sudarshana Santhosh Kumar Kothapalli, Zhiyao Yang, Xuwen Guo, Xiaowei Li, Yimin Cai, Wen Feng, Lihua Yuan

**Affiliations:** 1 College of Chemistry, Key Laboratory of Radiation Physics and Technology of the Ministry of Education, Institute of Nuclear Science and Technology, Sichuan University, Chengdu 610064, China; ywt1255751521@163.com (W.Y.); yangzhiyaohaha@sina.com (Z.Y.); xuwenguo94@126.com (X.G.); ymcai@scu.edu.cn (Y.C.); wfeng9510@scu.edu.cn (W.F.); 2 School of Sciences, Woxsen University, Hyderabad 502345, India; santhosh.kothapalli@gmail.com

**Keywords:** self-assembly, photoacid, hydrogen-bonded macrocycle, host–guest complexation

## Abstract

Achieving light-induced manipulation of controlled self-assembly in nanosized structures is essential for developing artificially dynamic smart materials. Herein, we demonstrate an approach using a non-photoresponsive hydrogen-bonded (H-bonded) macrocycle to control the self-assembly and disassembly of nanostructures in response to light. The present system comprises a photoacid (merocyanine, 1-MEH), a pseudorotaxane formed by two H-bonded macrocycles, dipyridinyl acetylene, and zinc ions. The operation of such a system is examined according to the alternation of self-assembly through proton transfer, which is mediated by the photoacid upon exposure to visible light. The host–guest complexation between the macrocycle and bipyridium guests was investigated by NMR spectroscopy, and one of the guests with the highest affinity for the ring was selected for use as one of the components of the system, which forms the host–guest complex with the ring in a 2:1 stoichiometry. In solution, a dipyridine and the ring, having no interaction with each other, rapidly form a complex in the presence of 1-MEH when exposed to light and thermally relax back to the free ring without entrapped guests after 4 h. Furthermore, the addition of zinc ions to the solution above leads to the formation of a polypseudorotaxane with its morphology responsive to photoirradiation. This work exemplifies the light-controlled alteration of self-assembly in non-photoresponsive systems based on interactions between the guest and the H-bonded macrocycle in the presence of a photoacid.

## 1. Introduction

The advent of a vast number of host–guest supramolecular structures significantly advances the growth in research productivity on stimuli-responsive systems for various applications such as functional nanoscale devices [1], photocontrolled catalysis [2], high-resolution patterning of surfaces with colloids [3], and molecular machines. Much effort has been devoted to supramolecular smart materials with reversible stimulus responsiveness that include pH [4], competitive binding, redox stimuli/inputs, light [5], and enzymes. Among these, controlled manipulation of the behavior of materials with pH stimulus boasts cheap acid–base reagents, fast operation, and wide options for proton supramolecular acceptors. However, the generation of waste products is unavoidable in the operation process, which constitutes a great huddle to the application of these materials. Light is appealing as the external stimulus in this context since it can be exploited spatiotemporally, noninvasively, with low energy consumption, and most importantly, without introducing additional chemical wastes. In most cases, photoactive moieties are anchored to material surfaces, such as nanoparticles, so as to render them responsive to light. This approach not only requires synthetic efforts but also deteriorates the performance of the switches owning to electronic interactions with metallic substrates [6,7]. In this regard, photoacids are particularly attractive because the photoinduced proton transfer or the pH change in closed systems can be regulated by external optical energy, as demonstrated in the implementation of molecular machines [8,9] and logic gates [10]. The easy operation of photoacids with light also benefits reversible assembly of supramolecular smart materials [11,12,13].

Responsive host–guest complexation associated with the use of photoacids and macrocycle has produced non-responsive systems with reversible light responsiveness. Elegant examples include non-photoresponsive rotaxanes that work in the presence of 1-MEH with a good fatigue resistance under visible light irradiation [14] and photoswitchable molecular shuttles without any photoresponsive group based on a photoinduced proton transfer (PIPT) strategy using an indazole-based photoacid [15]. The successful application of responsive host–guest systems relies heavily upon the option of macrocycles and functional components. (H-bonded) oligoamide macrocycles [16,17,18,19,20] particularly interesting for their demonstrated host–guest behaviors in supramolecular chemistry, as revealed in various applications such as recognition and extraction [21,22,23], separation [24], transmembrane channels [25], catalysis [26], and liquid-crystal materials [27], as well as forming mechanically interlocked molecules [28]. Different from many other two-dimensional shape-persistent macrocycles, these macrocycles of the smallest size have a cavity decorated with six carbonyl oxygen atoms pointing inward as binding sites. The electron-rich cavity renders it possible to bind cationic guest molecules such as dialkylammonium [29], tropylium [30], paraquat [31], and pyridinium [32]. We previously explored a photoswitchable host–guest system based on a non-photoresponsive H-bonded macrocycle and pyridinium for reversibly controlling the release and capture of the guest via proton transfer by a light stimulus with merocyanine 1-MEH [33]. However, the presence of only one potential recognition site (pyridinium) stymied further action for applications.

**Scheme 1:**
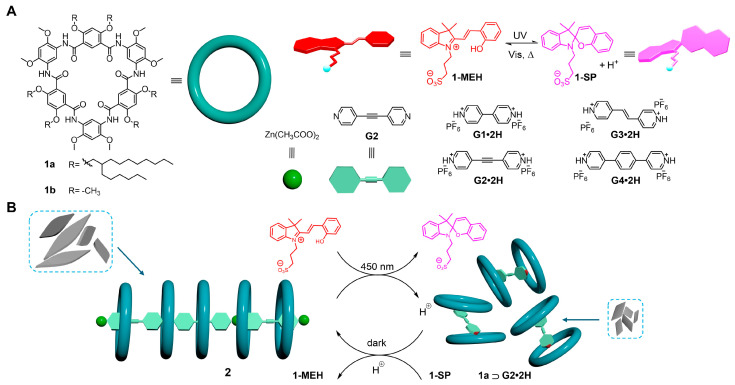
(**A**) Chemical structures of H-bonded macrocycle **1a** and guest molecules **G1·2H-G4·2H**. (**B**) Cartoon illustration of the photoacid 1-MEH controlled interconversion of self-assemblies based on polypseudorotaxane **2**. H-bonded macrocycle **1b** is used for DFT calculations due to structural simplicity.

Bipyridinium salts and their derivatives (e.g., viologen) are known to be excellent guests and well-recognized building units by coupling to macrocycles in supramolecular chemistry [34,35,36,37]. These guests feature reversible protonation (or deprotonation) due to the presence of two N-donor atoms that enable association and dissociation of the host–guest complexes through a chemically controlled acid–base reaction. Here, we describe a non-photoresponsive system that demonstrates the light-controlled reversible change in self-assembly of a supramolecular structure generated from polypseudorotaxane **2** in the presence of a photoacid (1-MEH). This is realized by subjecting dipyridinyl acetylene (**G2**)-zinc complex and macrocycle **1a** to protonation and deprotonation induced by 1-MEH under light irradiation, and the transformation of nano-rhombus to nano-lamellar suprastructures can be reversibly controlled with the photoacid via light-induced proton transfer (Scheme 1). The controlled self-assembly is evidenced by nuclear magnetic resonance (NMR), ultraviolet–visible (UV–vis) spectroscopy, dynamic light scattering (DLS), Fourier transform infrared (FT-IR) spectroscopy, and transmission electron microscopy (TEM).

The photoresponsive behavior of **G2** and macrocycle **1a** is found to be considerably different from pyridine and **1a** owing to the differentiated interplay of multiple interactions between the guest and the ring. So far, the use of a two-dimensional H-bonded macrocycle for controlling the interconvertible self-assembly of a non-photoresponsive suprastructure with light has not been reported.

## 2. Results and Discussion

### 2.1. Host–Guest Interaction

The feasibility of the complexation of host and guest is the first and foremost that needs to be tested before starting to research the prerequisite conditions for a workable self-assembling system. Inspired by our previous work on using H-bonded aramide macrocycle **1a** for recognition of pyridinium, we reasoned that bipyridinium and its analogous should be the right candidate for binding to the macrocycle. Four guests, **G1·2H-G4·2H,** were selected and prepared (Scheme S2) for subsequent binding experiments.

Screening experiments using NMR spectroscopy were carried out to examine the complexation ability of each guest for the host. This aims to find a suitable guest for constructing a light-controlled non-photoresponsive system not only with sufficiently high association constants but also with easy identification of signals in NMR spectra in a complicated medium, consisting of the host, the guest, and two isomers of photoacid in the solvent system tested. To this end, a 2 mM sample solution of each guest in a mixed solvent system comprising chloroform (CDCl_3_) and acetonitrile (CD_3_CN) (1:1, *v*/*v*) is subject to ^1^H NMR determination at ambient temperature. The first signal that suggests the interaction between the host and guests is the color change from faint to yellow upon mixing **1a** and **G1·2H-G4·2H** (Figure S2). This phenomenon is attributed to the formation of a charge transfer complex. In the cases of **G1·2H** and **G2·2H**, pronounced changes in chemical shifts of signals from both host and guest were observed (Figures S4 and S5), indicative of strong host–guest interactions between the macrocycle and these cationic guests.

**Figure 1:**
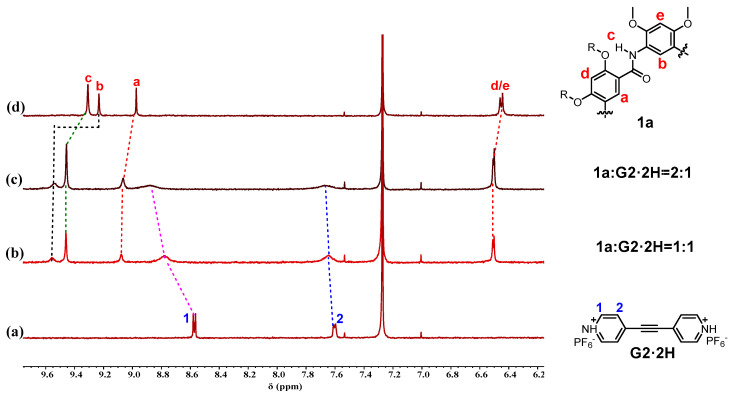
Partial ^1^H NMR spectra (400 MHz, CDCl_3_/CD_3_CN, 1:1, *v*/*v*, 298K) of (**a**) 1.0 mM **G2·2H**; (**b**) 1.0 mM **1a** and 1.0 mM **G2·2H**; (**c**) 2.0 mM **1a** and 1.0 mM **G2·2H**; (**d**) 1.0 mM **1a**.

Take a host–guest system comprising **G2·2H** and **1a**, for example, with two pyridinium moieties in **G2·2H** segregated by a triple bond spacer. Free guest **G2·2H** in CDCl_3_/CD_3_CN (1:1, *v*/*v*) show signals at 8.6 ppm and 7.6 ppm, respectively, while a 1:1 mixture of host **1a** and guest **G2·2H** (1.0 mM) presented signals (H^1^) that experience a pronounced downfield shift (Δδ = +0.35 ppm) relative to the proton resonance of free **G2·2H**, and the other signal (H^2^) is slightly shifted downfield (Δδ = +0.15 ppm). The change of chemical shifts in **G2·2H** indicates the complexation of **G2·2H** and the ring. On the other hand, a downfield shift of the aromatic protons (H^a^ and H^b^) in the host (Δδ = +0.10 ppm, Δδ = +0.31 ppm) is also an indication of the host–guest interaction. The response to the guest speaks for cation binding by the macrocycle and, thus, the formation of the host–guest complex. The process of complexation is found to be the fast exchange of the complex on the NMR timescale because a simple pattern of signals consisting of only a set of signals from the pyridinium ion and the macrocycle was observed upon addition of 2.0 eq. of **1a** to a 1.0 mM solution of **G2·2H** (Figure 1b and Figure S5). Results from HRESI mass spectrometry are in support of the strong affinity of the ring for **G2·2H,** where a peak of the highest intensity at *m*/*z* = 2428.4333, corresponding to [(**1a**)_2_ + **G2·2H** − 2PF_6_^−^]^2+^, is observed, indicating a 2:1 molar ratio for the complex (Figure S7). The same 2:1 stoichiometry was also observed for all other complexes formed in the gas state with guests **G1·2H**, **G3·2H**, and **G4·2H** in HRESI mass spectra (Figures S6–S9). This ratio is consistent with the results from Job plot experiments, which revealed 2:1 stoichiometry for all guests **G1·2H**-**G4·2H** (Figures S10–S13).

To pinpoint the specific binding site for the guest to reside in the complex, two-dimensional rotating frame nuclear Overhauser effect correlation spectroscopy (2D NOESY) of a 2:1 sample solution containing **1a** and **G2·2H** in CDCl_3_/CD_3_CN (1:1, *v*/*v*) was taken to acquire cross-peaks between signals from host and guest. The spectrum clearly shows the presence of cross signals (H^a^, H^2^), (H^a^, H^1^), and (H^b^, H^1^) between the signals attributable to the interior aromatic protons of **1a** (denoted as H^a^ and H^b^) and protons of **G2·2H** (denoted as H^1^ and H^2^), demonstrating the through-space interactions between **1a** and **G2·2H**. Since we failed to observe any correlations associated with the side-chain protons of the host and aromatic protons of the pyridinium of the guest (Figure S24), the binding for the guest is supposed to most likely occur in the macrocyclic cavity. Congruent with the NOESY results, DFT calculations reveal the presence of eight intermolecular H-bonding interactions and C-H···O interactions (Figure S37a). Independent gradient model (IGM) analysis (Figure S36b) revealed that the interring interaction in the **1a** + **G2·2H** complex (2:1) is sustained by π–π stacking interactions between two H-bonded macrocycles and C-H···O interactions between H-bonded macrocycles and bipyridinium. Therefore, the high affinity of the ring for the guest can be explained by the cooperative action of several weak interactions, including the dipole cation interaction, hydrogen bonding between the oxygen atoms of amide linkages, and pyridinium salts.

To quantify the binding affinity, UV–vis titration experiments were performed to determine the binding constants (Figures S16–S23). All guests show high binding affinities for the host in 1:1 CHCl_3_-CH_3_CN (Table 1), with **G2·2H** as the most powerful one. The binding affinity of **1a** for **G2·2H** was found to be (7.10 ± 0.53) × 10^9^ M^−2^ using the nonlinear curve fitting method (Figures S18 and S19). Interestingly, **G3·2H** and **G4·2H** afford the binding constant of (7.68 ± 0.18) × 10^5^ M^−2^ and (2.50 ± 0.03) × 10^8^ M^−2^ (Figures S21 and S23), respectively, which are more than one order of magnitude larger than the value for **G1·2H**.

Among the four guests, **G2·2H** exhibits higher cooperativity (α = 10.9) than those of all other three guests in binding the macrocycle. The difference in binding affinity observed can be ascribed to the outcome from steric hinderance (as in **G1·2H**) and electronic effects (as in **G3·2H** and **G4·2H**). For example, in the case of **G1·2H**, the two pyridinium units are so close to each other that repelling predominates between two rings, leading to diminished association as indicated by the lowest binding constant (2.70 × 10^5^ M^−2^). Inspection of the obtained ^1^H NMR spectra show that two key signals (H^1^ and H^2^) from aromatic protons of **G1·2H** and the macrocycle are overlapped, which would hinder the identification of the complexation process. As such, **G1·2H** is not considered for our later experiments, and only **G2·2H,** with the highest binding affinity and cooperativity, was opted for the following experiments (*vide infra*).

In a self-assembly system involving the use of photoacid, competitive binding from two or more guest species would make the complexation process complicated. Therefore, it is important for the photoacid in an ideal host–guest system to be devoid of any interaction with either the host (the macrocycle) or the guest (a neutral form of pyridinium salts) but only to protonate/deprotonate one of the components. Our prior work showed that macrocycle **1a** does not bind 1-MEH (photoacid), and there is no interaction between **1a** and the neutral form of pyridinium salts (guest). In this work, a different guest, bipyridinium, was employed. As such, we examined the interaction between **1a** and **G2**. When mixing the neutral form of **G2·2H** (i.e., **G2**) and host **1a** in a 1:2 molar ratio in CDCl_3_/CD_3_CN (1:1, *v*/*v*), the entire spectrum does not experience any change in signals and their chemical shifts (Figure S25), implying the absence of the complexation of the ring for the guest.

Therefore, the results above indicate that the macrocycle **1a** does not bind dipyridine, a neutral form of **G2·2H** in the course of light irradiation, and also would not interact with 1-MEH and its isomer in solution.

### 2.2. Photoisomerization Behavior of 1-MEH

Merocyanine [38] is known to function as a dynamic photoswitch for controlling host−guest complex [39,40]. In the literature, the solvent systems used vary from case to case, mostly focusing on protic solvents like water and methanol and polar aprotic solvents such as DMSO. The photoisomerization of 1-MEH pertains to the polarity of solvents [41]. Particularly, the conversion efficiency changed significantly with varying the solvent polarity. Thus, we first examined the photoisomerization behavior of 1-MEH in the selected solvent system CHCl_3_/CH_3_CN (1:1, *v*/*v*) before starting to investigate the reversible host–guest complexation.

**Figure 2:**
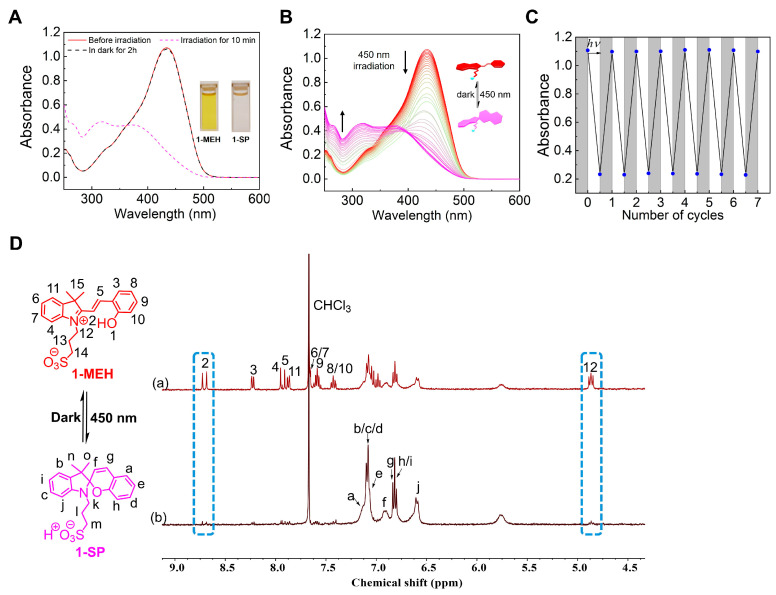
Photoisomerization and thermal relaxation of 1-MEH in a mixed solvent of CHCl_3_ and CH_3_CN (1:1, *v*/*v*). (**A**) UV–vis absorption spectra of 1-MEH (50 µM), ∼10 min after irradiation with 450 nm light (transformation into 1-SP), and after keeping the solution in the dark for 2 h after irradiation (reversion to 1-MEH); inset: photos of solutions of 1-MEH and 1-SP. (**B**) Stacked UV–vis absorption spectra of 1-MEH (50 µM), ∼10 min after irradiation with 450 nm light (transformation into **1-SP**); (**C**) Absorption at 435 nm upon irradiation/darkness cycling for 2 h in solution; (**D**) (**a**) ^1^H NMR (CDCl_3_/CD_3_CN, 1:1, *v*/*v*, 298 K, 400 MHz) spectrum of 1-MEH; (**b**) ^1^H NMR (CDCl_3_/CD_3_CN, 1:1, *v*/*v*, 298 K, 400 MHz) spectrum of 1-SP.

A solution of 1-MEH in CHCl_3_/CH_3_CN (1:1, *v*/*v*) (5 × 10^−5^ M) produces a yellowish solution which absorbs at λ_max_ = 435 nm in UV–vis absorption spectra (Figure 2A). This absorbance is similar to that observed for the analog of merocyanine bearing a butyl sulfonate group on the nitrogen of the indoline moiety in polar solvents such as acetone and water [42,43]. When exposed to visible light (450 nm), the absorption intensity of 1-MEH diminishes to its minimum in about 10 min and is regenerated again to its original value under dark in about 2 h (Figure 2B), demonstrating its complete reversibility. During light irradiation, an absorption at 245 nm appears, accompanied by the fading of the color of the solution, indicating the formation of the ring-closing isomer 1-SP along with the release of a proton to the solution. In the mixed solvent system selected, the phototransformation and thermal relaxation of 1-MEH/1-SP were highly reversible for seven cycles without any detectable fatigue (Figure 2C). The photoisomerization of 1-MEH was examined by ^1^H NMR spectroscopy in CHCl_3_/CH_3_CN (1:1, *v*/*v*). The spectra revealed the presence of 1-SP as the major product, with a sample solution being subject to exhaustive irradiation at 450 nm, suggesting the transformation from 1-MEH to 1-SP under this condition (Figure 2D). Such a result is consistent with the report from the literature [44].

### 2.3. Photoswitchable Complexation with Non-Photoresponsive Macrocycle

**Figure 3:**
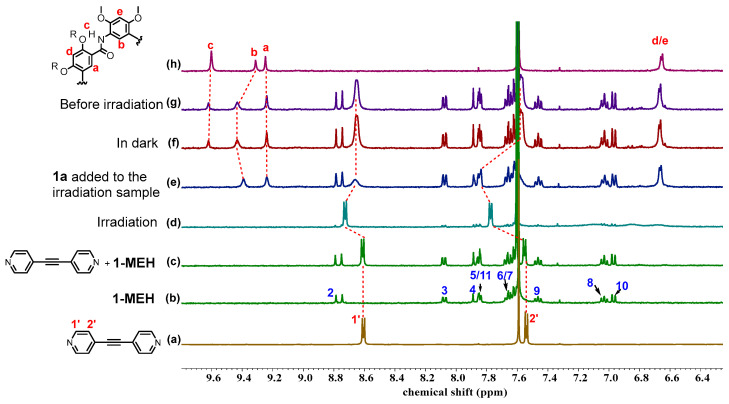
Stacked ^1^H NMR spectra (CDCl_3_/CD_3_CN, 1:1, *v*/*v*, 298 K, 400 MHz) of (**a**) 1,2-di(pyridin-4-yl) acetylene; (**G2**) (**b**) 1-MEH. (**c**) **G2** + 1-MEH (2.4 eq.) before irradiation; (**d**) solution from (**c**) irradiated for 10 min with 450 nm light; (**e**) **G2** + 1-MEH (2.4 eq.) + **1a** (2.0 eq.) irradiated for 10 min with 450 nm light; (**f**) solution from (**e**) kept under dark for 240 min; (**g**) **G2** + 1-MEH (2.4 eq.) + **1a** before irradiation; (**h**) H-bonded macrocycle **1a**. [**1a**]_0_ = [**G2**]_0_ = [1-MEH]_0_ = 1.0 mM.

To modulate a self-assembling system with non-photoresponsive components, it is essential to determine whether photoresponsive media, specifically solutions of photoacids, can effectively control the assembly. Protons H^1^′ and H^2^′ of **G2** exhibit a downfield shift of 0.10 ppm (Figure 3d), compared to the resonance frequency of the deprotonated form of **G2·2H** depicted in Figure 3a or its mixture prior to UV irradiation in Figure 3c. This shift indicates the protonation of **G2**, facilitated by the proton released from the photoacid.

It should be noted that 1-MEH releases a proton upon light irradiation, and the process is accompanied by the structural change in 1-MEH from a ring-closing form to spiropyran 1-SP, while the reverse process from 1-SP to 1-MEH occurs by absorbing a proton in the dark. Upon addition of 1 eq. of the macrocycle **1a** to the irradiated sample solution containing **G2·2H** and 1-SP, the signal from proton H^b^ undergoes a downfield shift (Δδ = +0.1 ppm) (Figure 3e) relative to the corresponding proton resonance of the macrocycle **1a** (Figure 3h), while protons H^1^ and H^2^ from **G2·2H** resonate upfield to a minor extent compared to those without the presence of the macrocycle **1a** (Figure 3d). These observations clearly indicate the complexation between the macrocycle and the positively charged guest. Under dark conditions, almost all the proton signals of the guest return to the previous position (Figure 3g) along with the reproduction of 1-MEH again because the reverse process of photoisomerization involves absorbing a proton in the dark through the transformation of 1-SP into 1-MEH, which converts the protonated **G2·2H** to the neutral form, leading to the release of the guest from the macrocycle **1a**. The complex dissociation occurs owing to the significantly reduced affinity of the macrocycle **1a** for the guest. These results confirm that the photoswitchable capture and release of guests by a non-photoresponsive host can be achieved with the present system comprising macrocycle **1a** and protonated Dipyridinyl acetylene **G2·2H**.

### 2.4. Controlled Reversible Polypseudorotaxane Formation Driven by Light

Having established a reversible photoswitchable host–guest system with non-photoresponsive H-bonded macrocycle **1a**, we reasoned that this system could be used for controlling the self-assembly of suprastructures by light irradiation (Scheme 1B).

**Figure 4:**
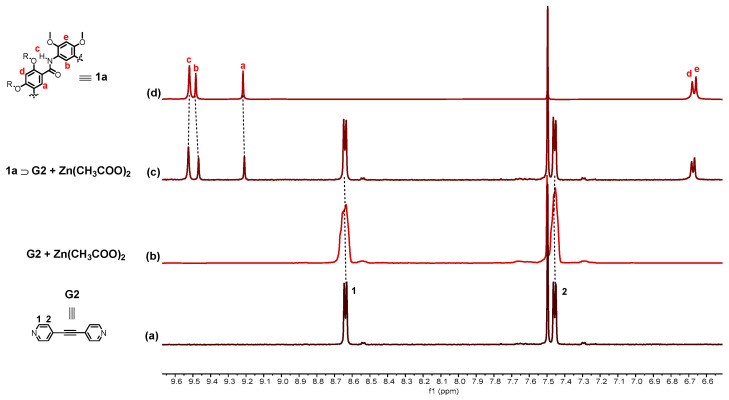
Stacked ^1^H NMR spectra (400 MHz, CDCl_3_/CD_3_CN, 1:1, *v*/*v*, 298 K) of (**a**) **G2** (1.0 mM); (**b**) **G2** (1.0 mM) and Zn(CH_3_COO)_2_ (2.0 mM); (**c**) **1a** (2.0 mM) and **G2** (1.0 mM) and Zn(CH_3_COO)_2_ (2.0 mM); (**d**) **1a** (1.0 mM).

To corroborate the interaction between **1a** and the coordination polymer formed by **G2** and zinc ion, ^1^H NMR experiments were carried out in CDCl_3_/CD_3_CN (1:1, *v*/*v*) with a sample solution containing **1a**, **G2**, and Zn^2+^. A 2:1:2 mixture of the macrocycle **1a**, **G2**, and zinc diacetate presented signals (H^1^ and H^2^) in two doublets that differ considerably from the broad signal pattern for the same protons of a mixture of **G2** and zinc salt. In addition, a minor shift for these two protons (Δδ = about 0.015 ppm) was observed with respect to the proton resonance of **G2** in the two-component mixture. On the other hand, a downfield shift of the aromatic protons (H^a^ and H^c^) (Δδ = 0.0064 ppm, Δδ = 0.0064 ppm) and an upfield shift of the aromatic protons (H^b^) (Δδ = 0.0349 ppm) in the macrocycle are also an indication of the host–guest interaction. These changes reflect cation binding by the macrocycle **1a** and thus the formation of the polypseudorotaxane (Figure 4).

**Figure 5:**
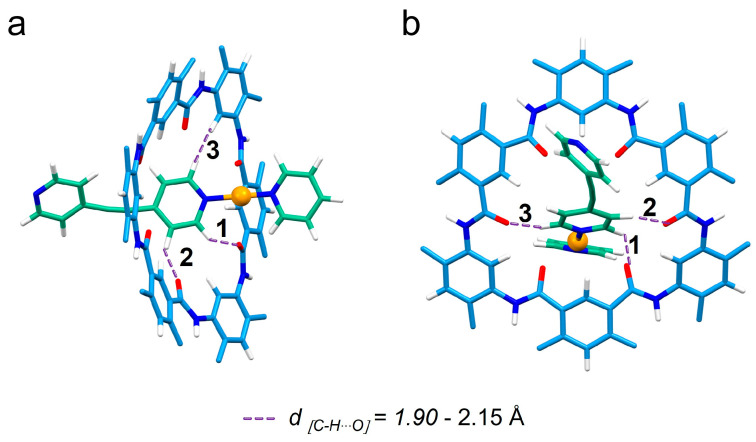
Optimized structure at the DFT/B3LYP (PCM, chloroform)/6-31G (d, p) level of complex **1a** ⊃ **G2** + Zn^2+^. (H—white, O—red, N—navy blue, C—blue and green, Zn—yellow). (**a**) Side view of the computational structure; (**b**) Top view of the computational structure; The dashed purple lines indicate C-H···O hydrogen bonds **1**–**3**, where **1** = 2.15 Å (133.7°), **2** = 1.96 Å (145.4°), and **3** = 1.90 Å (140.5°). All peripheral R groups are replaced by CH_3_ for simplicity.

The DFT calculations for **G2** + Zn^2+^ with macrocycle **1a** reveal a threaded supramolecular structure, evidenced by three hydrogen bonding interactions at distances ranging from 1.90 to 2.15 Å (Figure 5). Given the difficulty in obtaining the crystal structure of polypseudorotaxane **2**, we employed a mechanically interlocked model system to demonstrate the threading of **1a** onto a linear polymer chain. Inspired by our previous work, we synthesized the rotaxane in a “one-pot” process using macrocycle **1a** and **G2**. The successful synthesis resulted in a mixture of [3] rotaxane and [2] rotaxane, suggesting that a threaded structure consisting of the macrocycle and zinc-mediated polymer is plausible (Figures S29 and S30).

Upon comparing the Zn 2p, N 1s, and O 1s binding energies before and after the addition of **1a**, we observed shifts of +1.1 eV, −1.2 eV, and −2.1 eV, respectively. These changes suggest a modification in the chemical environment surrounding the Zn^2+^ coordination due to the formation of a three-component suprastructure **2** (Figure S31).

**Figure 6:**
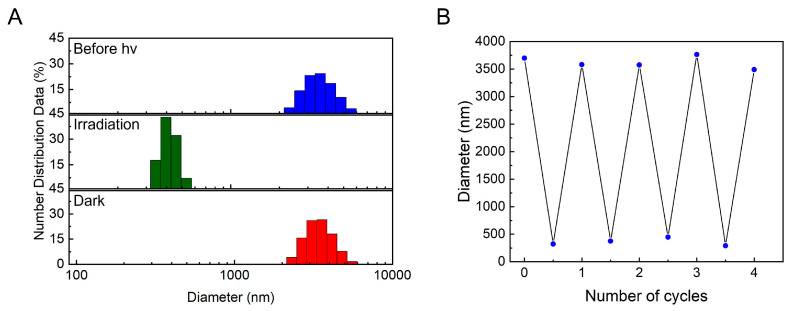
DLS of **1a** + **G2** + Zn^2+^ + 1-MEH: (**A**) Before irradiation; irradiate 10 min; and kept in the dark for 240 min after being irradiated; (**B**) number of cycles of DLS: alternate operation of exposing to irradiation and being in the dark for 240 min. [**G2**] = 25 µM. Solvent: CHCl_3_/CH_3_CN = 1:1 (*v*/*v*, 298 K).

Next, we further employed the dynamic light scattering (DLS) technique to confirm the reversible nature of the dissociation and association process of assemblies formed from polypseudorotaxane **2** in solution (Figure S32). Before irradiation, the average hydrodynamic diameter (D_h_) for a solution containing **1a**, **G2**, and 1-MEH is observed to fall in a size distribution of 3.60 μm, pointing to the formation of polypseudorotaxane **2** (Figure 6A, before *hv*). After irradiation at 450 nm for 10 min, the D_h_ of the aggregates is significantly reduced to 400 nm. We attribute this change to the protonation-induced disruption of the assemblies owing to the presence of 1-MEH. Transmission electron microscopy (TEM) images revealed the presence of leaf-like nano-objects of about 4 μm from the samples containing polypseudorotaxane **2** before irradiation, but the assemblies break down to small strip bulks of about 400 nm after irradiation (Figure S33). The results from TEM experiments about the morphology of the aggregated samples are consistent with those from DLS experiments.

Interestingly, under dark conditions for 240 min, the assemblies increase to the size with the D_h_ of 3.60 μm (Figure 6A (dark)), indicating the reversibility of the assembly process. The present system works better in four interconvertible assembly–disassembly cycles, as shown by DLS measurement (Figure 6B). It should be noted that disassembly only proceeds to some extent, as indicated by the difference in size change before irradiation and after being kept in the dark, which is consistent with the results from TEM experiments.

**Figure 7:**
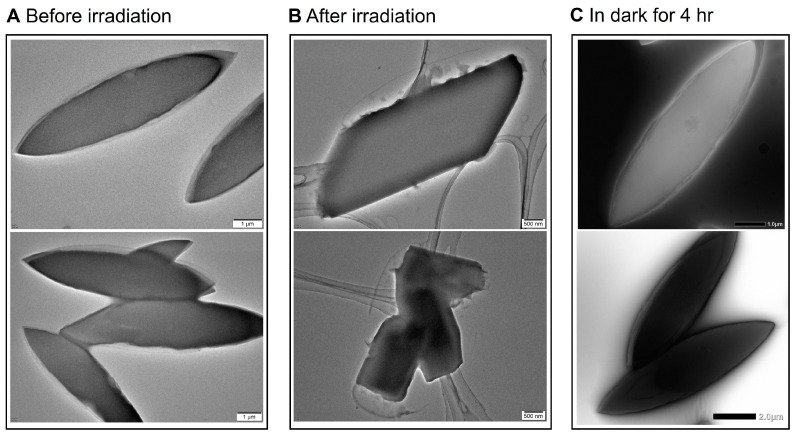
TEM of **1a** (2.0 eq.) + **G2** + Zn^2+^(2.0 eq.) + 1-MEH (2.4 eq.): (**A**) **1a** (2.0 eq.) + **G2** + Zn^2+^ (2.0 eq.) + 1-MEH (2.4 eq.) before 450 nm irradiation; (**B**) **1a** (2.0 eq.) + **G2** + Zn^2+^ (2.0 eq.) + 1-MEH (2.4 eq.) with 450 nm irradiate 10 min; (**C**) **1a** (2.0 eq.) + **G2** + Zn^2+^ (2.0 eq.) + 1-MEH (2.4 eq.) kept in the dark for 240 min after being irradiated at 450 nm for 10 min. [**G2**] = 25 µM. (CHCl_3_/CH_3_CN = 1:1, *v*/*v*, 298K).

TEM images of the polypseudorotaxane **2** without irradiation reveal a nano-rhombus suprastructure (Figure 7A) but change into a nano-lamellar suprastructure upon irradiation for 10 min (Figure 7B). When placed in the dark, it returns to the original morphology (Figure 7C).

The results above indicate that coupling the host–guest system to 1-MEH and zinc ions produces a controlled assembly system of light-switchable nano-aggregates that are otherwise impossible on its own.

## 3. Materials and Methods

### 3.1. Materials and Reagents

4,4′-Bipyridine, 1,2-di (pyridin-4-yl) acetylene (**G2**), 1,2-dipyridine ethylene, 1,4-bis (pyrid-4-yl) benzene, HCl (conc.), NH_4_PF_6_, CH_3_CH_2_OH, K_2_CO_3_, H_2_SO_4_ (conc.), and CDCl_3_ were purchased from Energy Chemical (Chengdu, China). CD_3_CN was purchased from Energy Chemical (Anhui, China). HPLC CHCl_3_ and CH_3_CN were purchased from Chron Chemicals (Chengdu, China). All reagents purchased from commercial suppliers were used without further purification.

### 3.2. Experimental Methods

^1^H NMR spectra were recorded on Bruker AVANCE AV II-400/600 MHz at room temperature of 298 K (^1^H: 400 MHz; 2D: 600 MHz) (Bruker, Karlsruhe, Germany). The 2D NOESY spectra were acquired using an AV II-600 MHz nuclear magnetic resonance spectrometer in CDCl_3_/CH_3_CN = 1:1, *v*/*v* at 298 K (Bruker, Karlsruhe, Germany). Host–guest stoichiometries were obtained from ESI-HRMS spectra acquired on LCMS-IT-TOF (SHIMADZU, Kyoto, Japan). Stoichiometries based on Job plot experiments and binding constants with UV–vis spectra were measured by SHIMADZU UV-2450 (Kyoto, Japan). The binding constants were obtained by nonlinear fitting with a method named Nelder–Mead on the website (supramolecular.org, accessed on 7 May 2024). The changes in UV–vis spectra at 365 nm were plotted along with the added guest equivalent. Then, the obtained plots were fitted with the nonlinear curve method by selecting the option “UV 2:1.”

### 3.3. Synthesis of Hydrogen-Bonded Macrocycle 1a and Guests G1·2H-G4·2H

Macrocycles **1a** and guests **G1·2H-G4·2H** were synthesized according to previous references [45,46,47] (Schemes S1 and S2, Figures S1 and S2).

### 3.4. DFT Calculations

DFT calculations for the geometrical optimizations were performed in Gaussian 09 program package. All substituents (R) on the periphery of macrocycle **1a** are replaced by methyl groups to give compound **1b** for simplicity.

### 3.5. Visualization of Noncovalent Interactions

Independent gradient model (IGM) analysis is an approach [45] based on promolecular density (an electron density model prior to molecule formation) to identify and isolate intermolecular interactions. DFT-optimized structures are used as input files. Strong polar attractions, van der Waals contacts, and repulsive forces are visualized as an isosurface with blue, green, and red colors, respectively. The binding surface was calculated by the Multiwfn 3.8 program [46] and visualized using PyMOL version 1.7 [48].

## 4. Conclusions

In summary, we have presented the use of a non-responsive hydrogen-boned macrocycle **1a** for controlling the reversible transformation of nanosized suprastructures with visible light. Such an approach involves the coupling of the host–guest complexation to the photoacid 1-MEH and coordination with zinc ions in the solution. The polypseudorotaxane can be dissociated and rebuilt through controlling irradiation with 450 nm light. This work provides a supplementary entry to the light-controlled self-assembly of non-photoresponsive suprastructures with H-bonded macrocycles and holds the potential for constructing other supramolecular systems that do not rely on photoresponsive constituents for applications.

## Figures and Tables

**Table 1:** Association constants (*K*_a_) for complexation of various guests (**G1·2H-G4·2H**) by host **1a** at 298 K ^a^.

Guest	CHCl_3_/CH_3_CN	*K*_1_ (M^−1^)	*K*_2_ (M^−1^)	*K*_a_ (M^−2^)	α ^b^
**G1·2H**	1:1	(2.05 ± 0.18) × 10^4^	(1.30 ± 0.06) × 10^4^	(2.70 ± 0.06) × 10^8^	2.5
**G2·2H**	1:1	(1.61 ± 0.03) × 10^4^	(4.41 ± 0.25) × 10^4^	(7.10 ± 0.53) × 10^9^	10.9
**G3·2H**	1:1	(8.06 ± 0.17) × 10^2^	(9.53 ± 0.03) × 10^2^	(7.68 ± 0.18) × 10^5^	4.7
**G4·2H**	1:1	(1.81 ± 0.08) × 10^4^	(1.38 ± 0.04) × 10^4^	(2.50 ± 0.03) × 10^8^	3.0

^a^ The association constant *K*_a_ values were obtained by UV–vis titration experiments; the concentration of **1a** was fixed at 50 µM. ^b^ α represents the cooperativity factors defined as α = 4*K*_2_/*K*_1_.

## Data Availability

Data are contained within the article and Supplementary Materials.

## Supplementary Materials

The following supporting information can be downloaded at: https://www.mdpi.com/article/10.3390/molecules29204842/s1, Scheme S1: synthetic route of **1a**, Scheme S2: synthetic routes of guests **G1·2H-G4·2H**; Figure S1: ^1^H NMR spectrum of **1a**; Figure S2: The color changes in host **1a** and the guest guests **G1·2H-G4·2H** are mixed; Figure S3: ^1^H NMR spectra of 1-MEH and 1-SP isomerization; Figures S4–S7: ^1^H NMR spectra of **1a** and **G1·2H-G4·2H** interactions; Figures S8–S11: HRMS spectrum of **G1·2H-G4·2H** ⊂ **1a**; Figures S12–S15: Job plot for determination of stoichiometry of **G1·2H-G4·2H** ⊂ **1a**; Figures S16–S23: UV–vis titration experiments and complex constant of macrocycle **1a** and guest **G1·2H-G4·2H**; Figure S24: 2D NOESY spectrum for **G2·2H** ⊂ **1a**; Figure S25: ^1^H NMR spectra for **1a** and neutral form of **G2·2H** interactions; Figure S26: ^1^H NMR spectra for **1a** and **G2** interactions with 1-MEH to 1-SP; Figure S27: Staked ^1^H NMR spectra of **1a** + **G2** + Zn^2+^; Figure S28: DFT calculations of **1a** ⊃ **G2** + Zn^2+^; Figure S29: ESI-HRMS of [3]rotaxane and [2]rotaxane; Figure S30: ^1^H NMR spectra of [3]rotaxane and [2]rotaxane; Figure S31: XPS of **1a** + **G2** + Zn^2+^ and **G2** + Zn^2+^; Figure S32: Number of cycle of DLS of **1a** + **G2·2H** + Zn^2+^ + 1-MEH; Figure S33: TEM of **1a** + **G2** + Zn^2+^ + 1-MEH; Figure S34: TEM of **G2** + Zn^2+^ + 1-MEH; Figures S35–S38: DFT calculation studies for **1a** and **G1·2H-G4·2H.** References [49,50] are cited in the Supplementary Materials.
