# Supplementary material for: Light-Controlled Interconvertible Self-Assembly of Non-Photoresponsive Suprastructures
Source: Molecules. 2024 Oct 12;29(20):4842. doi: 10.3390/molecules29204842 (PMC11509933; doi:10.3390/molecules29204842)
Supplement: Supplementary file 1 [file molecules-29-04842-s001.zip › molecules-3124947-supplementary.pdf]

Supporting Information

Light-Controlled Interconvertible Self-Assembly of Non-Photoresponsive Suprastructures

Wentao Yu [1,‡], Sudarshana Santhosh Kumar Kothapalli [2,‡], Zhiyao Yang [1], Xuwen Guo [1], Xiaowei Li [1,*], Yimin Cai [1], Wen Feng [1] and Lihua Yuan [1,*]

[1] College of Chemistry, Key Laboratory of Radiation Physics and Technology of the Ministry of Education, Institute of Nuclear Science and Technology, Sichuan University, Chengdu 610064, China;

[2] School of Sciences, Woxsen University, Telangana - 502345, India;

ywt1255751521@163.com (W.Y.); santhosh.kothapalli@gmail.com (S.K.); yangzhiyaoha-ha@sina.com (Z.Y.); 782695553@qq.com (X.G.); ymcai@scu.edu.cn (Y.C.); wfeng9510@scu.edu.cn (W.F.)

‡ These authors contributed equally to this work.

** Correspondence: lhyuan@scu.edu.cn (L.Y.); lixw@scu.edu.cn (X.L.)*

Table of Contents

Synthesis and characterization……………………………………………………………..3-5

Host-guest charge transfer complex……………………………………………………..…..…5

¹H NMR spectra of **1-MEH** and **1-SP** isomerization………………………..……….…..…..6

Host-guest chemistry of macrocycle **1a** and **G1•2H-G4•2H**……………………………..…7-8

HRMS spectrum of **G1•2H-G4•2H** ⊂ **1a**..9-10

Job plot for determination of stoichiometry of **G1•2H-G4•2H** ⊂ **1a**................…..........11-12

UV-vis titration experiments of macrocycle **1a** and guests **G1•2H-G4•2H**.....................13-16

2D NOESY spectrum of **G2•2H** ⊂ **1a**..17

¹H NMR spectra for 1a and neutral form of **G1•2H**...17

¹H NMR spectra for **1a** ,**1-MEH** and **G2**..18

Stacked ¹H NMR spectra of **1a** + **G2** + Zn^{2+}..19

DFT calculations of **1a** ⊃ **G2** + Zn^{2+}…………………………..…..………...………… 20-25

ESI-HRMS of [3]rotaxane and [2]rotaxane 26

¹H NMR spectra of [3]rotaxane and [2]rotaxane..27

XPS of **1a** + **G2** + Zn^{2+} and **G2** + Zn^{2+}...28

Number of cycles of DLS **1a** + **G2•2H** + Zn^{2+} + **1-MEH**..28

TEM of **1a** + **G2** + Zn^{2+} + **1-MEH**...29

TEM of **G2** + Zn^{2+} + **1-MEH**...29

DTF calculations of **1a** and guest **G1•2H-G4•2H**………………..……………..………….30-54

References..…………..…………..…………..……………..……..…………..…………………….55

Synthesis and characterization

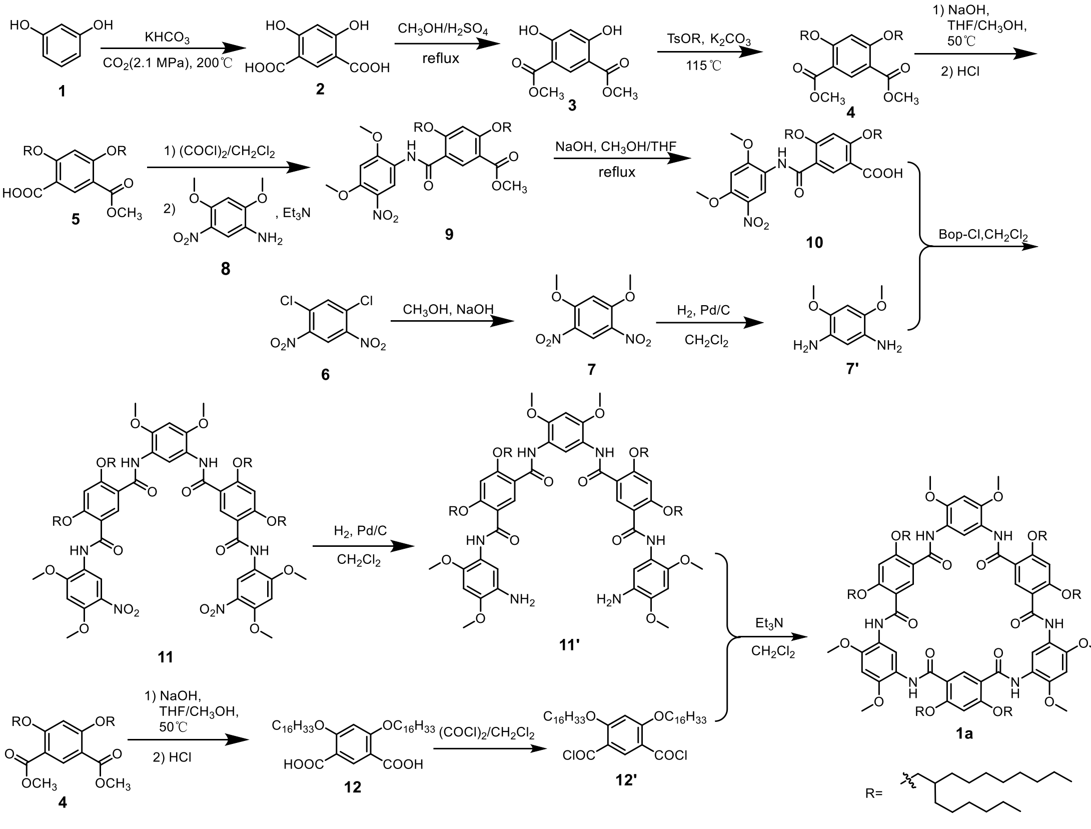

Scheme S1 Synthetic route of **1a**.

1a was prepared according to literature procedures.[1]

1a: white solid powder (yield: 64 %). ^{1}H NMR (400 MHz, $CDCl_3$, 298 K): δ 9.98 (*s*, 2H), 9.75 (*s*, 4H), 9.24 (*d*, *J* = 2.6 Hz, 4H), 7.99 (*dd*, *J* = 8.7, 2.7 Hz, 4H), 7.09 (*d*, *J* = 8.8 Hz, 4H), 6.53 (*s*, 2H), 4.15 (*d*, *J* = 6.3 Hz, 8H), 3.91 (*s*, 12H), 2.12 (*p*, *J* = 6.2 Hz, 4H), 1.63–1.15 (*m*, 108H), 0.85 (*tt*, *J* = 6.8, 3.3 Hz, 24H).

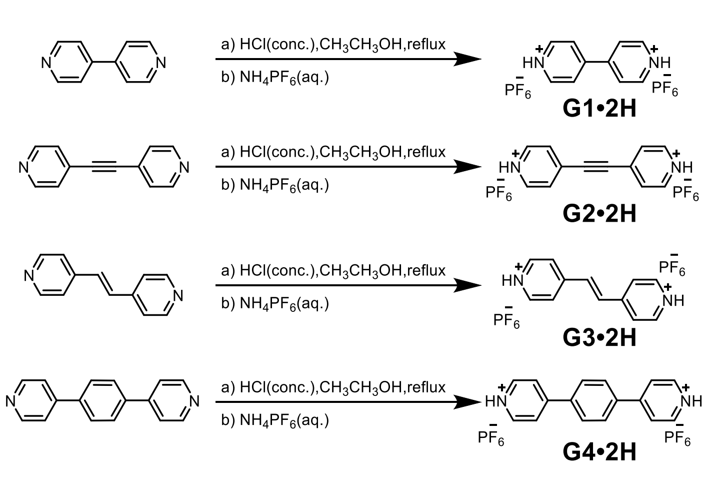

Scheme S2 Synthetic routes of guests **G1•2H-G4•2H**.

Guest **G1•2H**[2]

To a solution of 4,4'-bipyridine (235 mg, 1.50 mmol) in EtOH (5.0 mL), HCl (conc.) (25 mL) was added. The mixture was stirred for overnight at 80 ℃. The reaction mixture was added cold EtOH (100 mL) under ice bath after being cooled to room temperature. The mixture was filtered and washed with cold EtOH (10 mL × 3). The solid was dissolved in water. The saturated aqueous solution of NH_4PF_6 was slowly added to the solution above and the mixture was stirred for 0.5 hr. After filtration, the precipitate was collected and washed with H_2O. After being dried in vacuum for 24 h, guest **G1•2H** (430 mg, 48%) was obtained as a white powder. ^{1}H NMR (400 MHz, CD_3CN, 298 K) δ 9.13–9.06 (*m*, 4H), 8.38–8.32 (*m*, 4H).

Guest **G2•2H**[3]

To a solution 1,2-di(pyridin-4-yl) acetylene (300 mg, 1.66 mmol) in EtOH (5.0 mL), HCl (conc.) (25 mL) was added. The mixture was stirred for overnight at 80 ℃. The reaction mixture was added cold EtOH (100 mL) under ice bath after being cooled to room temperature. The mixture was filtered and washed with cold EtOH (10 mL × 3). The solid was dissolved in water. The saturated aqueous solution of NH_4PF_6 was slowly added to the solution above and the mixture was stirred for 0.5 hr. After filtration, the precipitate was collected and washed with H_2O. After being dried in vacuum for 24 h, guest **G2•2H** (393 mg, 50%) was obtained as a white powder. ^{1}H NMR (400 MHz, CD_3CN, 298 K) δ 8.86 – 8.79 (m, 4H), 8.35 (d, J = 6.3 Hz, 4H), 4.38 (s, 6H).

G3•2H-G4•2H were prepared according to similar procedure of **G2•2H**.

Guest **G3•2H** white solid powder (yield: 48 %). [4] ^{1}H NMR (400 MHz, CD_3COCD_3, 298 K) δ 9.76 (*s*, 2H), 9.13 – 9.06 (*m*, 4H), 8.38 – 8.32 (*m*, 4H).

Guest **G4•2H** white solid powder (yield: 46 %). ^{1}H NMR (400 MHz, DMSO-d_6) δ 9.02 – 8.91 (*m*, 4H), 8.48 – 8.31 (*m*, 4H), 8.24 (*d*, *J* = 17.4 Hz, 4H).

^1H NMR spectra of the compounds

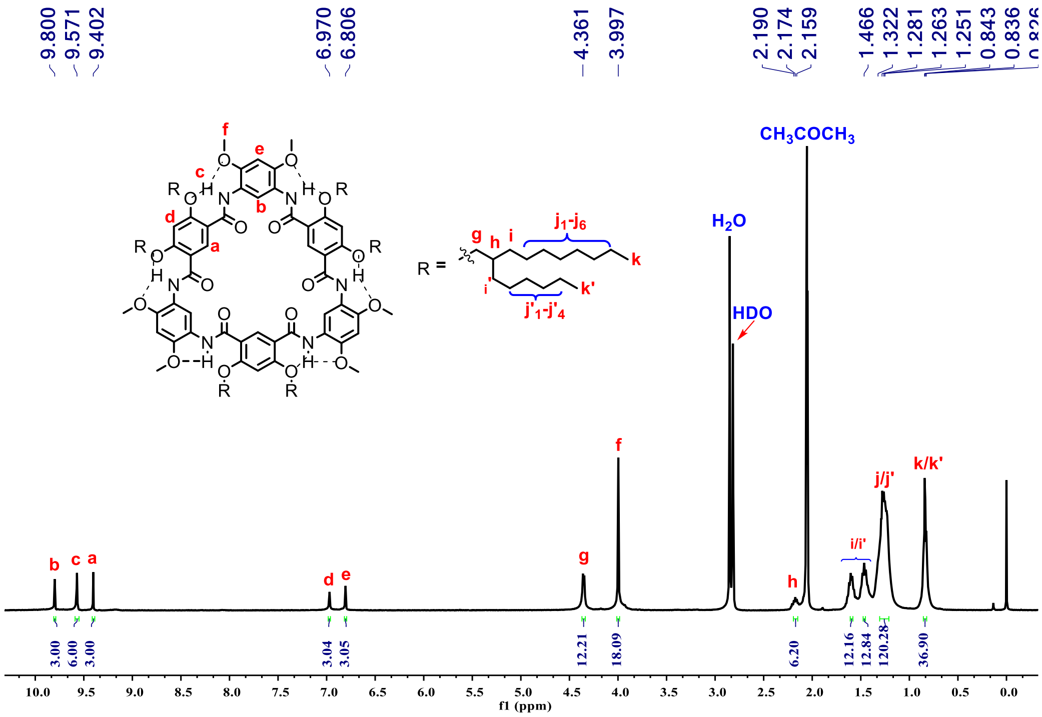

Figure S1 ^1H NMR spectrum (400 MHz, *acetone-d_6*, 298 K) of **1a**.

Host-guest charge transfer complex

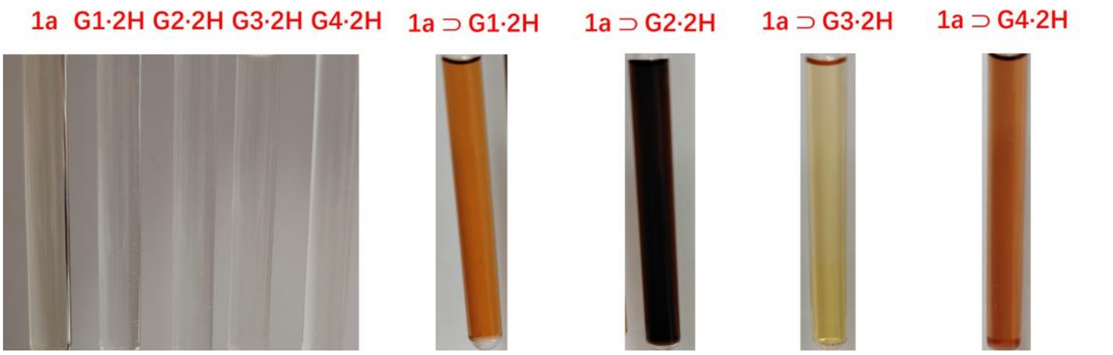

Figure S2 The images of guest solutions of **G1•2H-G4•2H** and the host-guest complexes solutions of **1a** ⊃ **G1•2H**, **1a** ⊃ **G2•2H, 1a** ⊃ **G3•2H,** and **1a** ⊃ **G4•2H**, showing color changes caused by charge transfer interactions.

1-MEH and **1-SP** isomerization

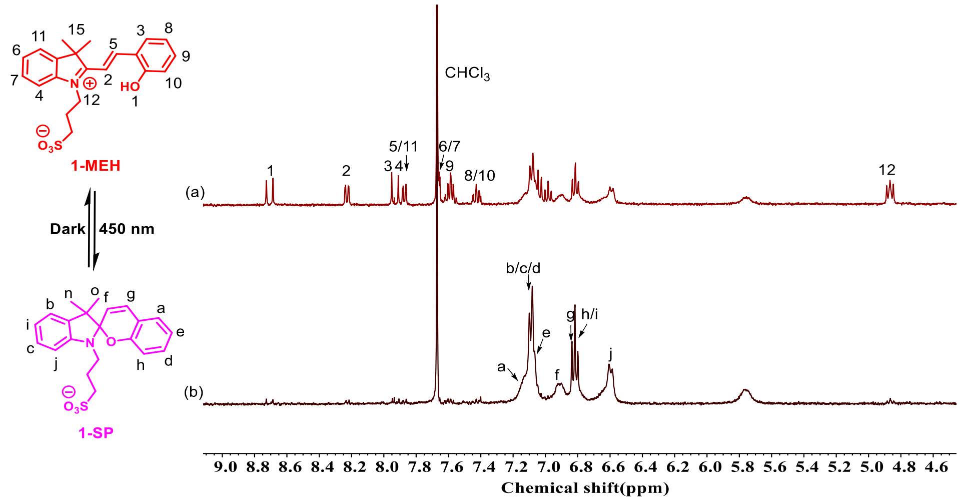

Figure S3 ^{1}H NMR spectra ($CDCl_3/CD_3CN$, 1:1, *v/v*, 298 K, 400 MHz) of **1-MEH** and **1-SP** isomerization.

Host-guest chemistry of macrocycle **1a** and **G1•2H-G4•2H**

^{1}H NMR spectra for **1a** and **G1•2H-G4•2H** interactions

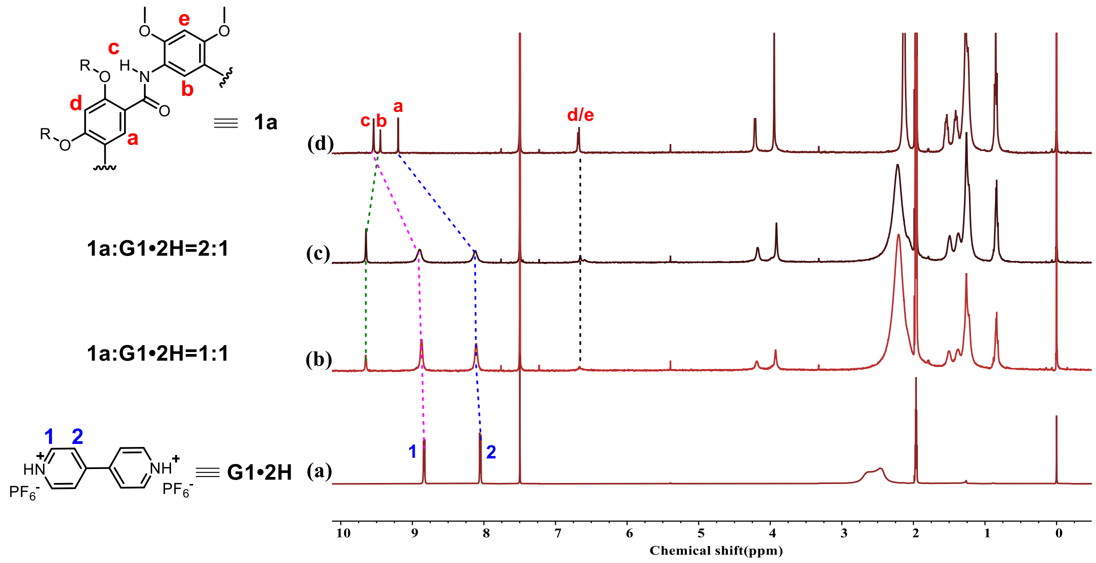

Figure S4 ^{1}H NMR spectra (400 MHz, $CDCl_3/CD_3CN$, 1:1, *v/v*, 298K) of (a) 1.0 mM **G1•2H**, (b) 1.0 mM **1a** and 1.0 mM **G1•2H**, (c) 2.0 mM **1a** and 1.0 mM **G1•2H**, (d) 1.0 mM **1a**.

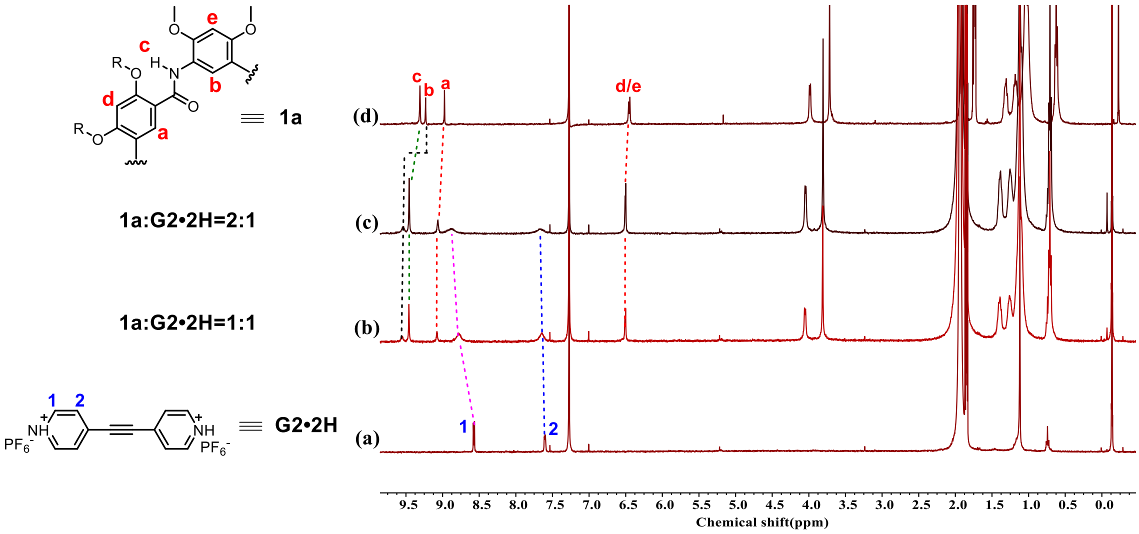

Figure S5 ^{1}H NMR spectra (400 MHz, $CDCl_3/CD_3CN$, 1:1, *v/v*, 298K) of (a) 1.0 mM **G2•2H**, (b) 1.0 mM **1a** and 1.0 mM **G2•2H**, (c) 2.0 mM **1a** and 1.0 mM **G2•2H**, (d) 1.0 mM **1a**.

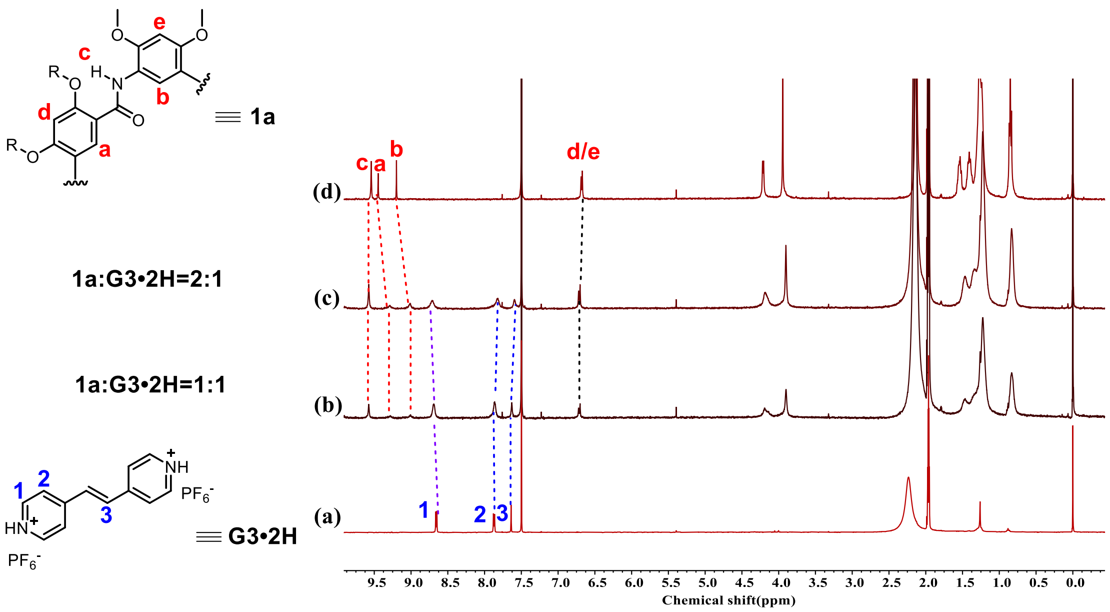

Figure S6 ^{1}H NMR spectra (400 MHz, $CDCl_3$/CD_3CN, 1:1, *v/v*, 298K) of (a) 1.0 mM **G3•2H**, (b) 1.0 mM **1a** and 1.0 mM **G3•2H**, (c) 2.0 mM **1a** and 1.0 mM **G3•2H**, (d) 1.0 mM **1a**.

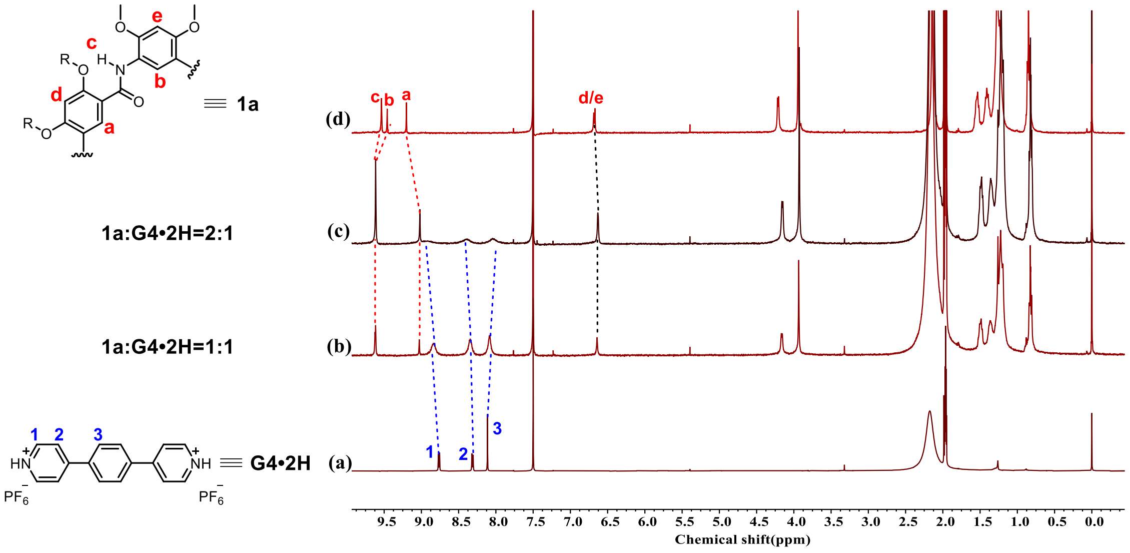

Figure S7 ^{1}H NMR spectra (400 MHz, $CDCl_3$/CD_3CN, 1:1, *v/v*, 298K) of (a) 1.0 mM **G4•2H**, (b) 1.0 mM **1a** and 1.0 mM **G4•2H**, (c) 2.0 mM **1a** and 1.0 mM **G4•2H**, (d) 1.0 mM **1a**.

MALDI-TOF-MS spectra of host-guest complexes

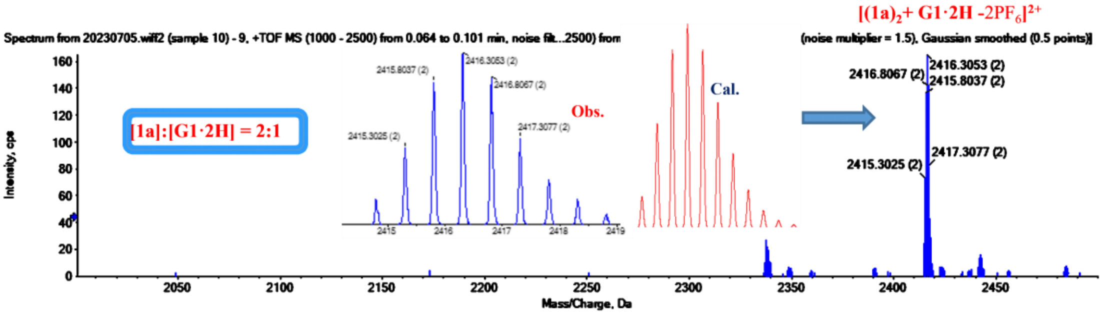

Figure S8 MALDI-TOF-MS of **1a** ⊃ **G1 • 2H** complex in $CHCl_3/CH_3CN$ (1:1, *v/v*). The calculated (blue) and experimental (red) isotopic distribution for [(**1a**)$_2$ + **G2•2H** - $2PF_6^-]^{2+}$, m/z, 2416.3047, found 2416.3053.

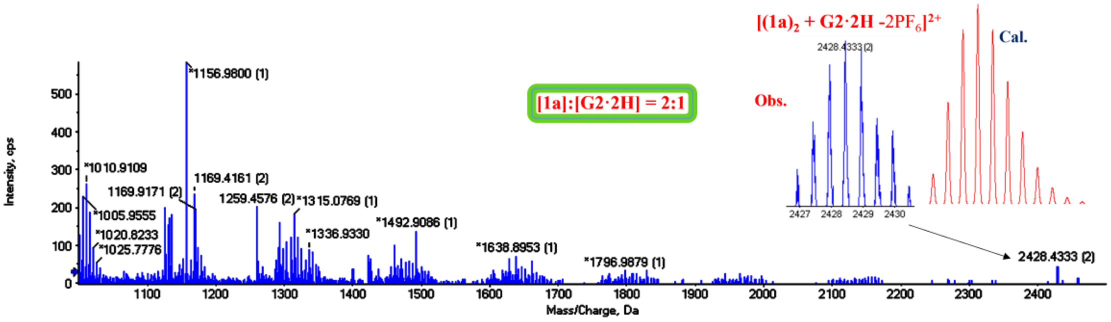

Figure S9 MALDI-TOF-MS of **1a** ⊃ **G2 • 2H** complex in $CHCl_3/CH_3CN$ (1:1, *v/v*). The calculated (blue) and experimental (red) isotopic distribution for [(**1a**)$_2$ + **G2•2H** - $2PF_6^-]^{2+}$, m/z, 2428.3047, found 2428.4333.

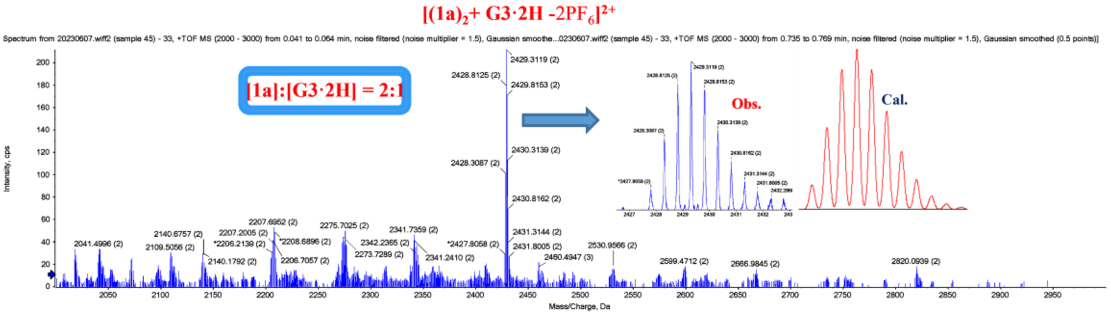

Figure S10 MALDI-TOF-MS of **1a** ⊃ **G3 • 2H** complex in $CHCl_3$-CH_3CN 1:1, *v/v*). The calculated (blue) and experimental (red) isotopic distribution for [(**1a**)$_2$ + **G3•2H** - $2PF_6^-]^{2+}$, m/z, 2429.3125, found 2429.3119.

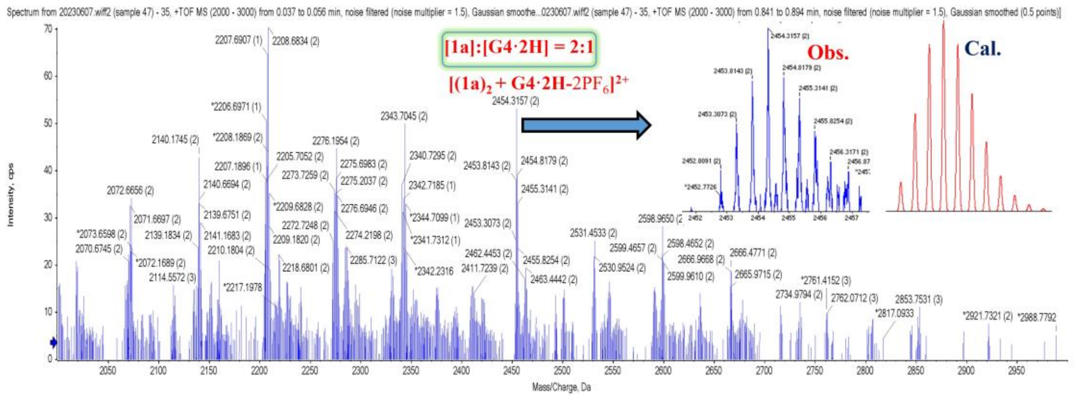

Figure S11 MALDI-TOF-MS of **1a** ⊃ **G4 • 2H** complex in $CHCl_3$-CH_3CN 1:1, *v/v*). The calculated (blue) and experimental (red) isotopic distribution for [(**1a**)$_2$ + **G4•2H** - 2PF_6^-]$^{2+}$, m/z, 2454.3203, found 2454.3157.

Job plot for the determination of stoichiometry of host-guest complexes

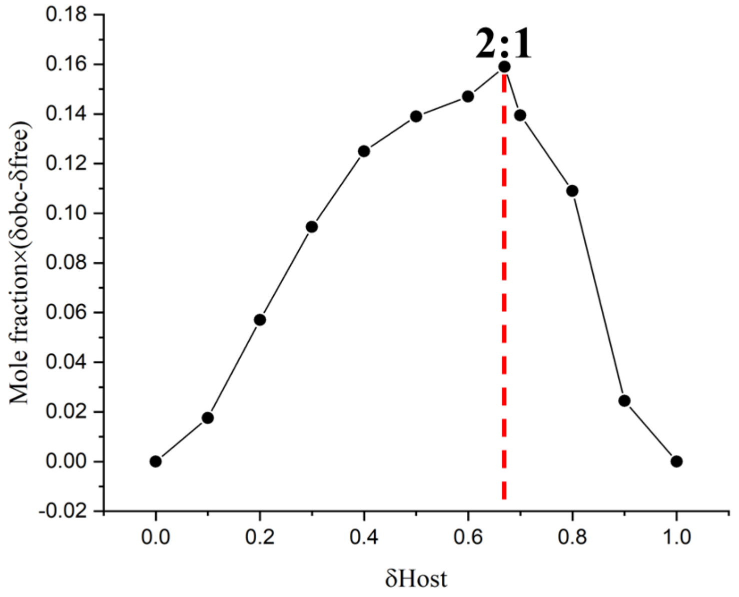

Figure S12 Job plot for the determination of stoichiometry of **1a** and **G1•2H** based on the absorbance at 365 nm in $CHCl_3/CH_3CN$ (1:1, *v/v*, 298 K). The total concentration of [**1a**] and [**G1•2H**] is 50 μM.

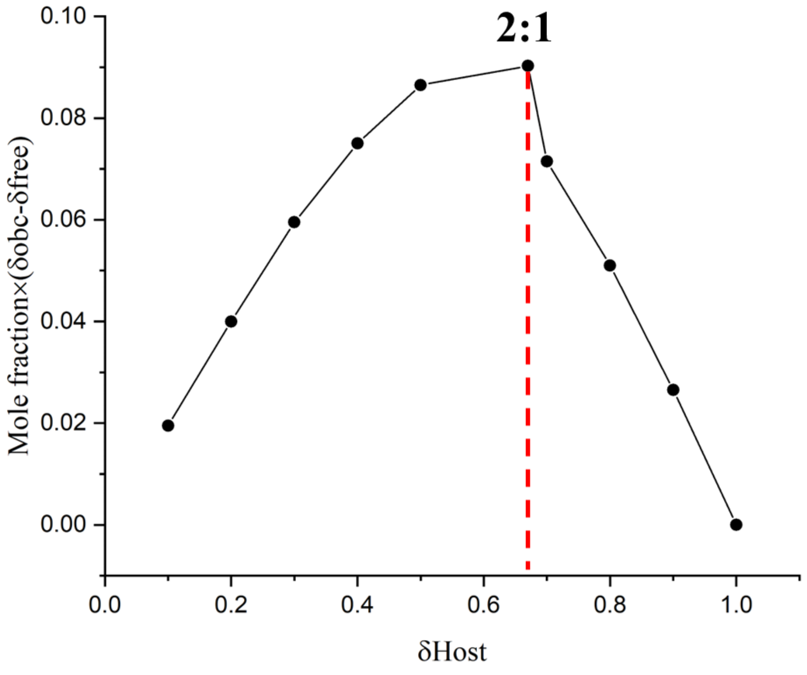

Figure S13 Job plot for the determination of stoichiometry of **1a** and **G2•2H** based on the absorbance at 365 nm in $CHCl_3/CH_3CN$ (1:1, *v/v*, 298 K). The total concentration of [**1a**] and [**G2•2H**] is 50 μM.

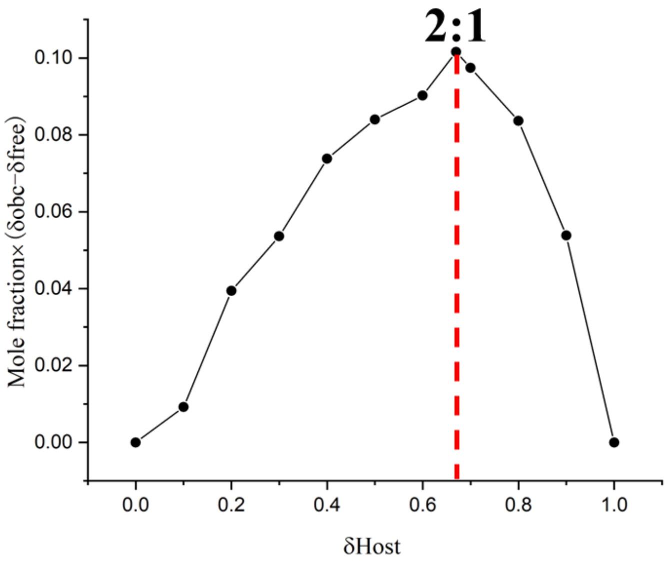

Figure S14 Job plot for the determination of stoichiometry of **1a** and **G3•2H** based on the absorbance at 365 nm in $CHCl_3/CH_3CN$ (1:1, *v/v*, 298 K). The total concentration of [**1a**] and [**G3•2H**] is 50 μM.

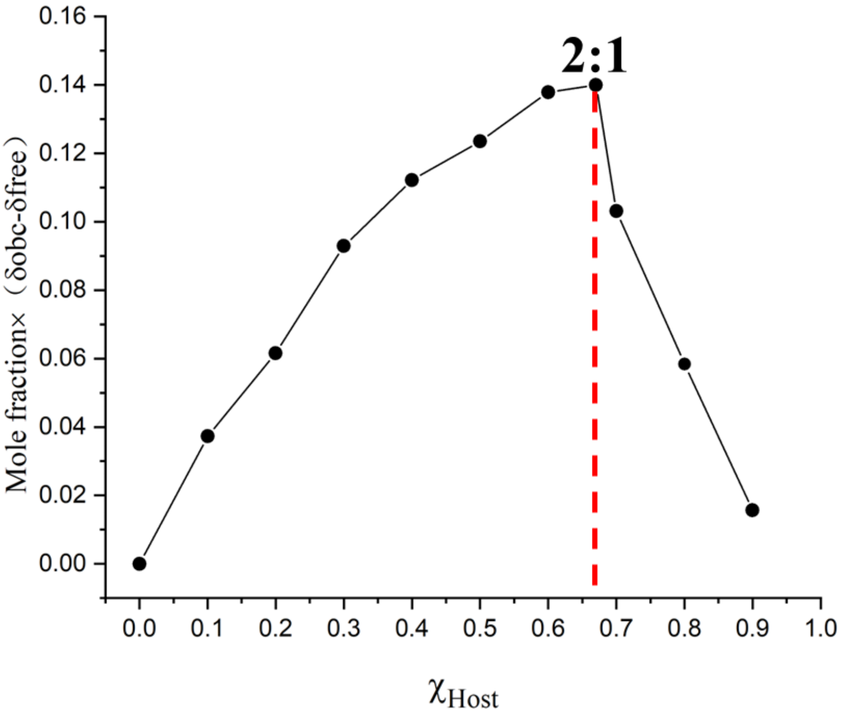

Figure S15 Job plot for the determination of stoichiometry of **1a** and **G4•2H** based on the absorbance at 365 nm in $CHCl_3/CH_3CN$ (1:1, *v/v*, 298 K). The total concentration of [**1a**] and [**G4•2H**] is 50 μM.

UV-vis titration experiments of macrocycle **1a** and guests **G1•2H-G4•2H**

To determine the binding constant (K_a) of macrocycle **1a** and guest **G1•2H-G4•2H**, UV-vis titration experiments were performed in $CHCl_3$-CH_3CN (1:1, v/v, 298 K) at a constant concentration of **1a** (50 μM) and varying concentration of **G1•2H-G4•2H**. For the titration, at least 20 data points were collected. Binding constant was calculated by a global fitting analysis according to a 2:1 binding model using the website *(http://supramolecular.org/)*.

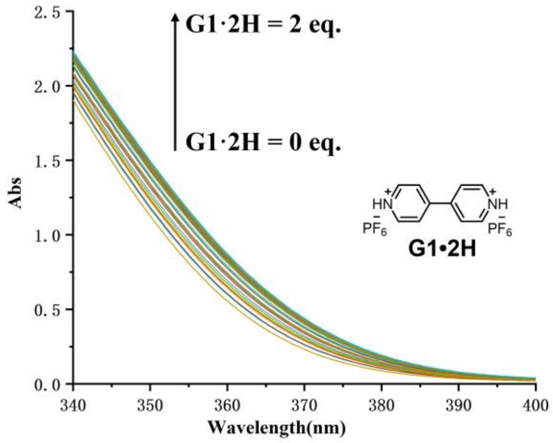

Figure S16 Stacked UV-vis spectra of **1a** (50 μM) titrated with **G2•2H** from 0 equiv. to 2.0 equiv. in solution ($CHCl_3$/CH_3CN, 1:1, *v/v*, 298 K).

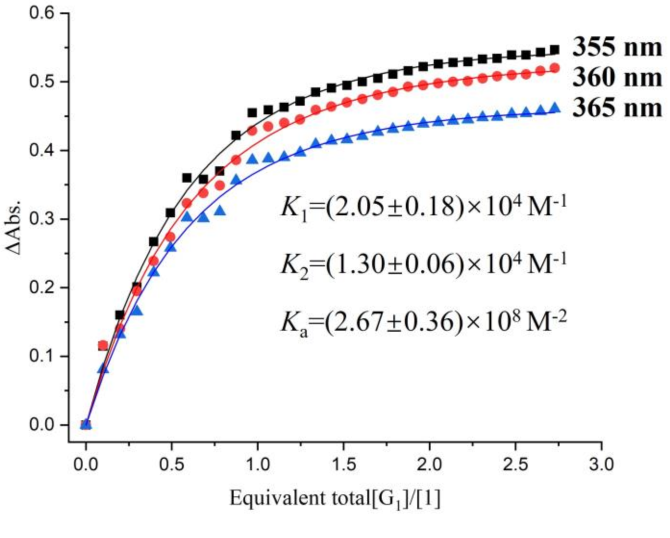

Figure S17 Curve fitting of the binding constant of **G1•2H** ⊂ **1a** in solution ($CHCl_3$/CH_3CN, 1:1, *v/v*, 298 K). The reported binding constant is the average value based on fitting of the absorbance at 355 nm, 360 nm, and 365 nm.

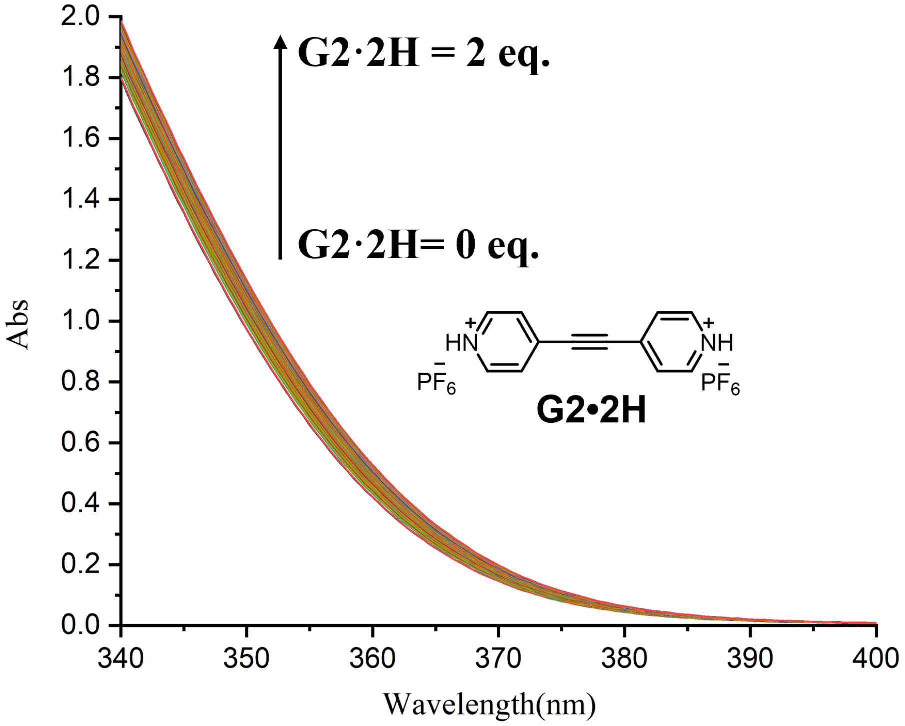

Figure S18 Stacked UV-vis spectra of **1a** (50 μM) titrated with **G2 • 2H** from 0 equiv. to 2.0 equiv. in solution ($CHCl_3/CH_3CN$, 1:1, *v/v*, 298 K)..

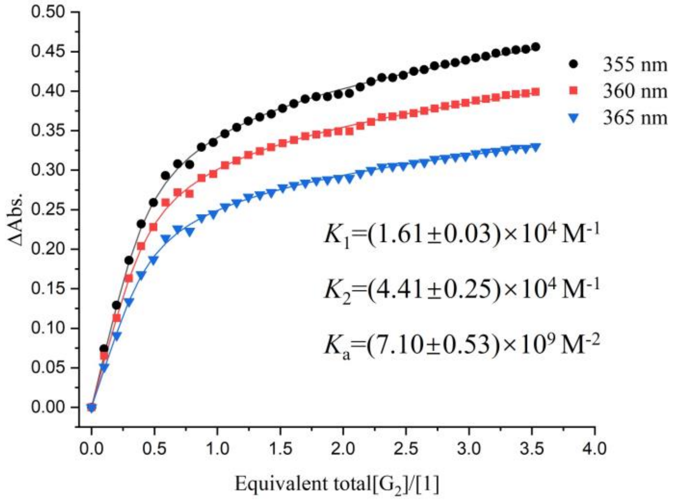

Figure S19 Curve fitting of the binding constant of **G2•2H** ⊂ **1a** in solution ($CHCl_3/CH_3CN$, 1:1, *v/v*, 298 K).. The reported binding constant is the average value based on fitting of the absorbance at 355 nm, 360 nm, and 365 nm.

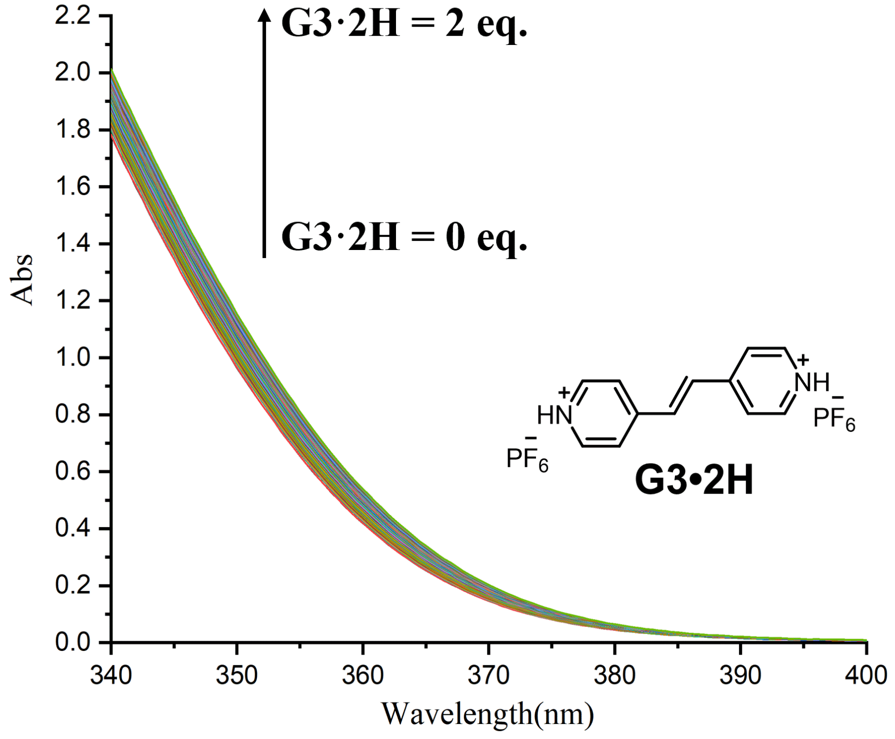

Figure S20 Stacked UV-vis spectra of **1a** (50 μM) titrated with **G3•2H** from 0 equiv. to 2.0 equiv. in solution ($CHCl_3$/CH_3CN, 1:1, *v/v*, 298 K).

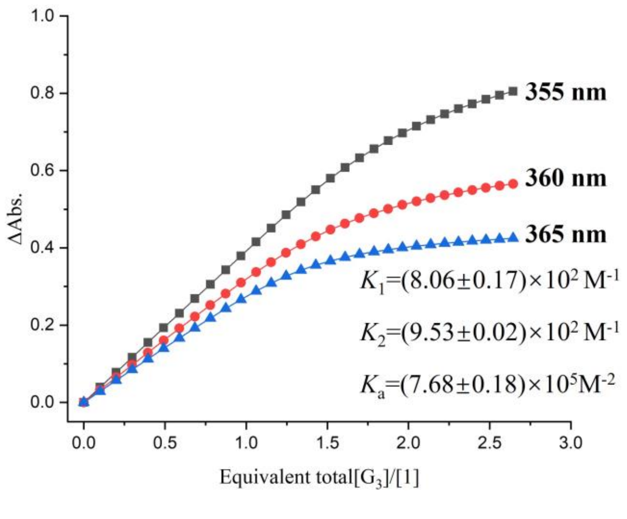

Figure S21 Curve fitting of the binding constant of **G3•2H** ⊂ **1a** in solution ($CHCl_3$/CH_3CN, 1:1, *v/v*, 298 K). The reported binding constant is the average value based on fitting of the absorbance at 355 nm, 360 nm, and 365 nm.

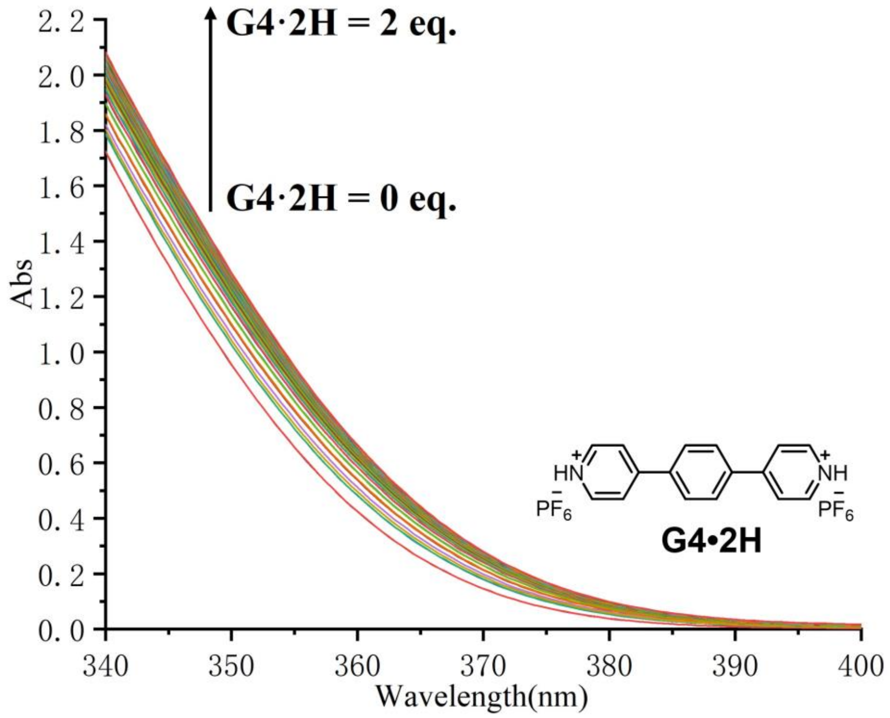

Figure S22 Stacked UV-vis spectra of **1a** (50 μM) titrated with **G4•2H** from 0 equiv. to 2.0 equiv. in solution ($CHCl_3/CH_3CN$, 1:1, *v/v*, 298 K).

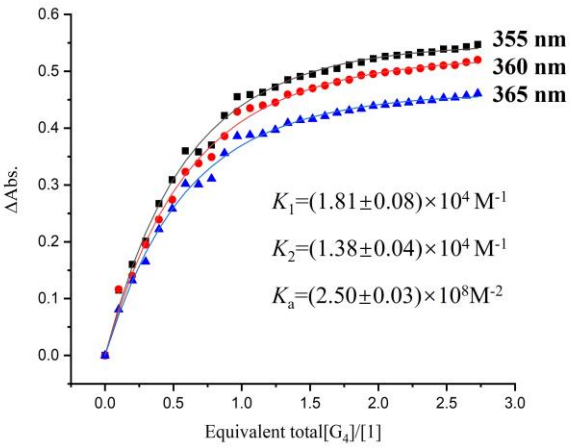

Figure S23 Curve fitting of the binding constant of **G4•2H** ⊂ **1a** in solution ($CHCl_3/CH_3CN$, 1:1, *v/v*, 298 K). The reported binding constant is the average value based on fitting of the absorbance at 355 nm, 360 nm, and 365 nm.

2D NOESY spectrum of **G2•2H ⊂1a**

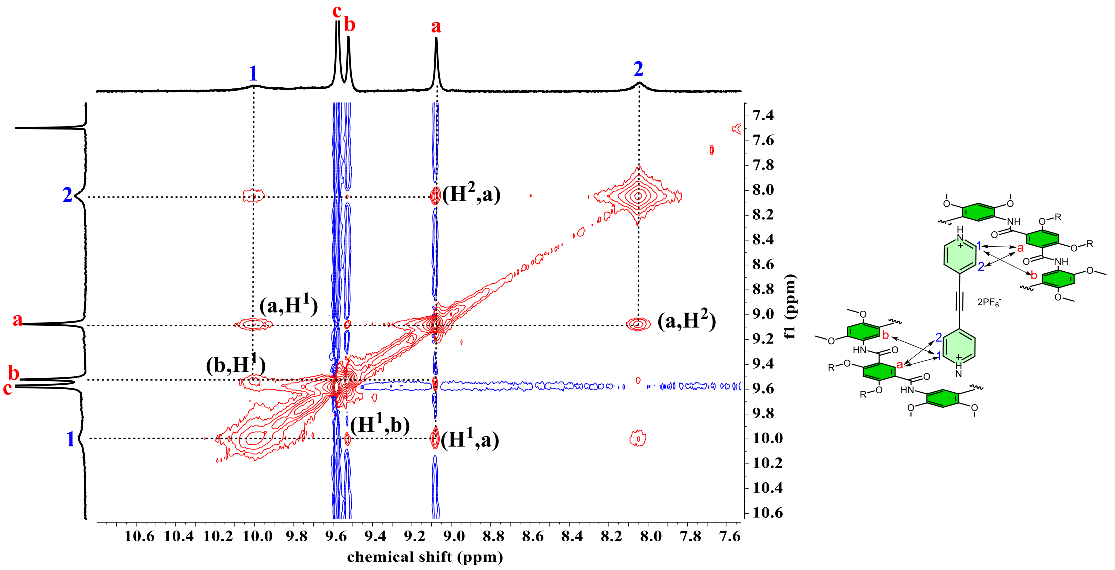

Figure S24 Expanded 2D-NOESY spectrum of **1a** ⊃ **G2•2H** ([**1a**] = 10 mM, [**G2•2H**] = 5 mM, **1a : G2•2H** = 2:1) (600 MHz, $CDCl_3$-CD_3CN, 1:1, *v/v*, 298 K, mixing time=0.4 s).

^{1}H NMR spectra for **1a** and neutral form of **G2•2H** interactions

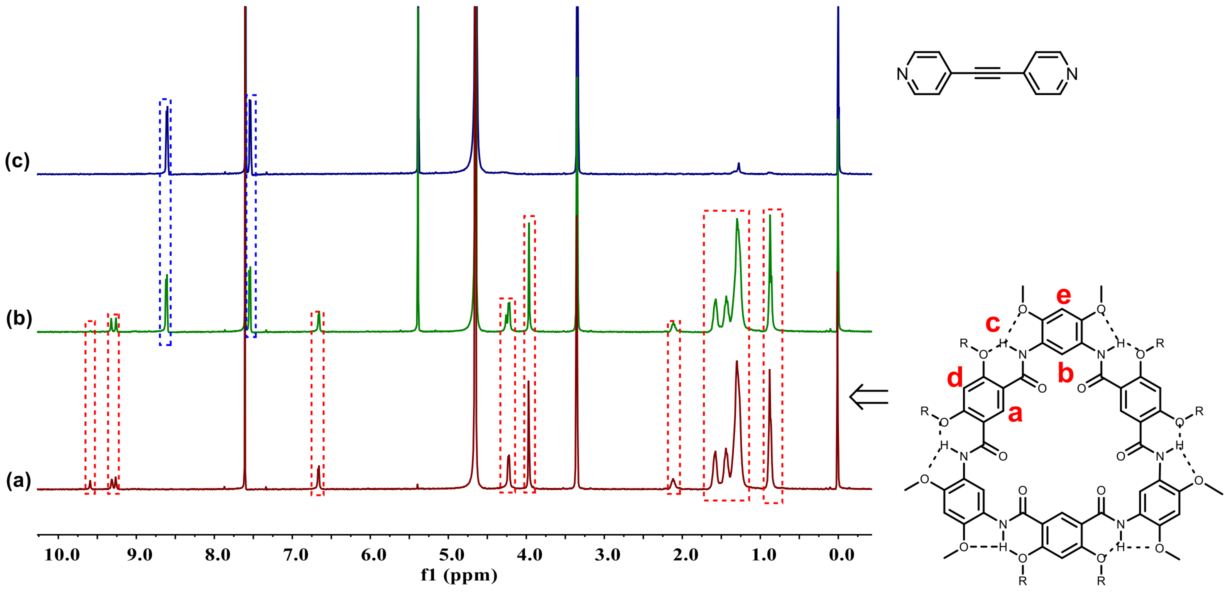

Figure S25 ^{1}H NMR spectra (400 MHz, $CDCl_3/CD_3CN$, 1:1, *v/v*, 298K) of (a) 1.0 mM **1a**, (b) 1.0 mM **1a** and 1.0 mM 1,2-di(pyridin-4-yl) acetylene, (c) 1.0 mM **1a** and 1.0 mM 1,2-di(pyridin-4-yl) acetylene.

^{1}H NMR spectra for host-guest chemistry of system

The lamp source is positioned 50 cm from the solution to facilitate observation of the isomerization process by slowing it down.

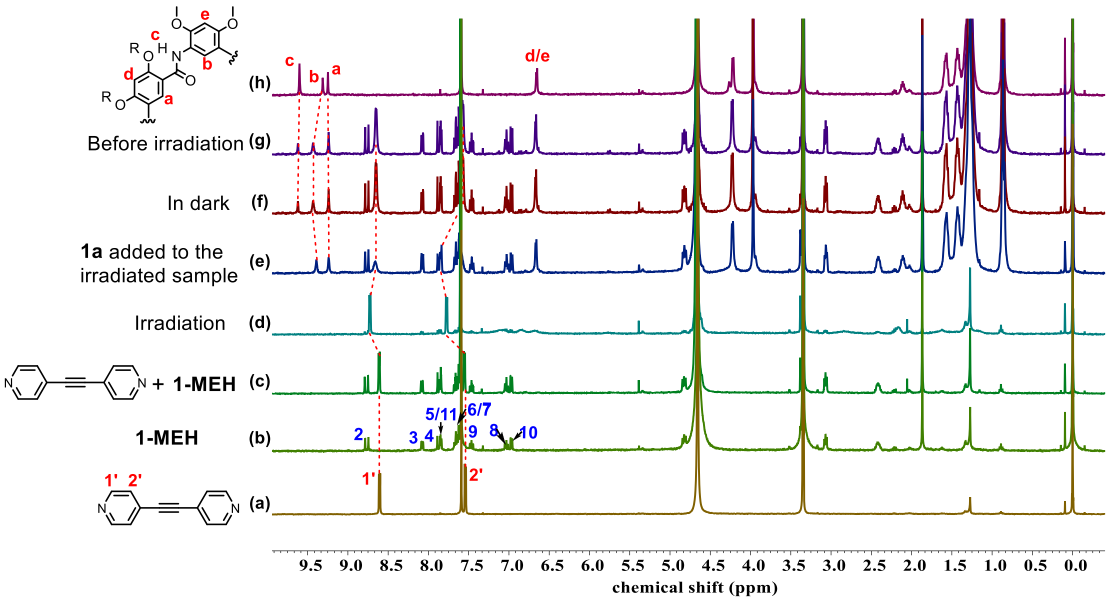

Figure S26 Stacked ^{1}H NMR spectra ($CDCl_3/CD_3CN$, 1:1, *v/v*, 298 K, 400 MHz) of (a) 1,2-di(pyridin-4-yl) acetylene (**G2**). (b) **1-MEH**. (c) **G2** + **1-MEH** (2.4 equiv.) before irradiation. (d) solution from (c) irradiated for 10 min with 450 nm light. (e) **G2** + **1-MEH** (2.4 equiv.) + **1a** (2.0 equiv.) irradiated for 10 min with 450 nm light. (f) solution from (e) kept under dark for 240 min. (g) **G2** + **1-MEH** (2.4 equiv.) + **1a** before irradiation. (h) hydrogen-bonded macrocycle **1a**. $[\mathbf{1a}]_0$ = $[\mathbf{G2}]_0$ = $[\mathbf{1\text{-}MEH}]_0$=1.0 mM.

Stacked ^{1}H NMR spectra of **1a + G2 +** Zn^{2+}

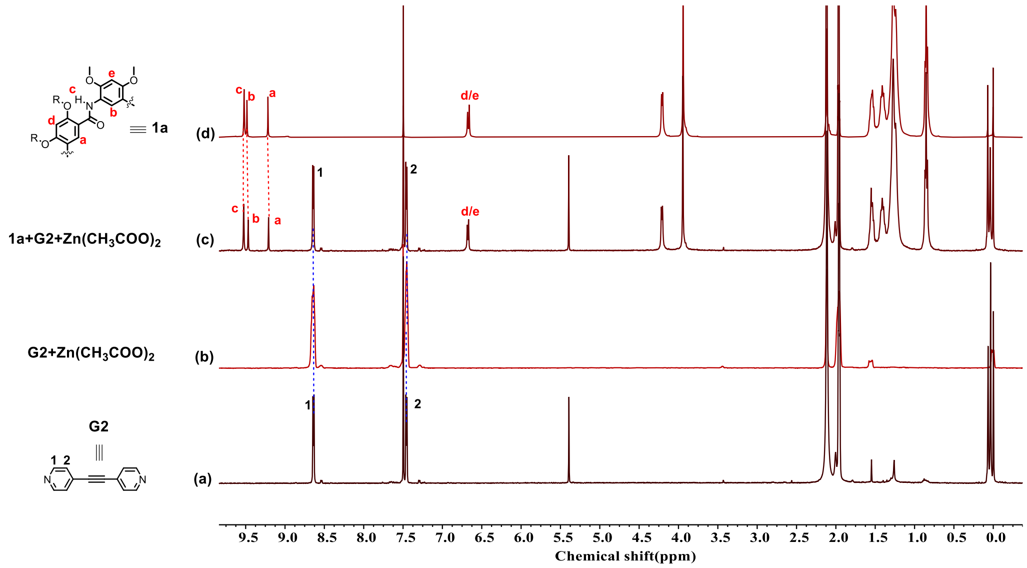

Figure S27 Staked ^{1}H NMR spectra (400 MHz, $CDCl_3$-CD_3CN, 1:1, *v/v*, 298 K) of (a) **G2** (1.0 mM), (b) **G2** (1.0 mM) and $Zn(CH_3COO)_2$(2.0 mM), (c) **1a** (2.0 mM) and **G2** (1.0 mM) and $Zn(CH_3COO)_2$ (2.0 mM), (d) **1a** (1.0 mM).

DFT Calculations of **1a** ⊃ **G2** + Zn^{2+}

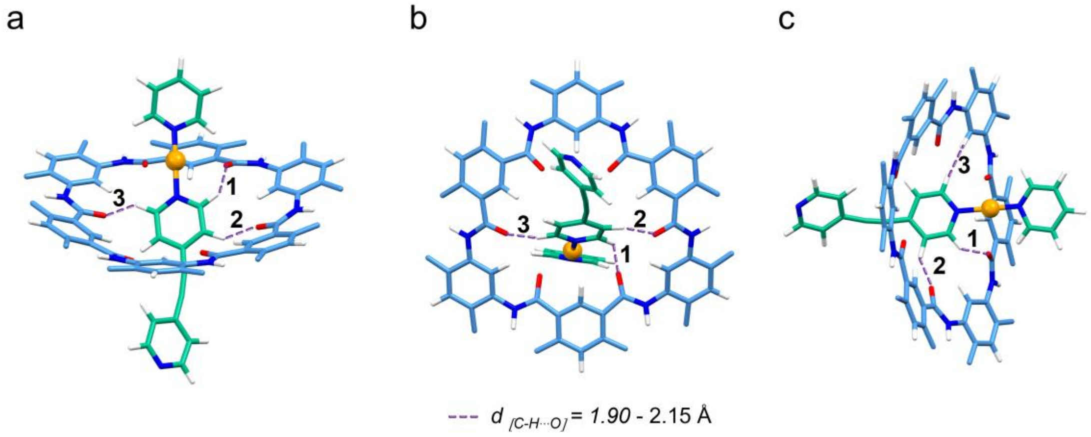

Figure S28 Optimized structure at the DFT/B3LYP(PCM, chloroform)/6-31G (d,p) level of complex **1a ⊃ G2 +** Zn^{2+}. (H—white, O—red, N—navy-blue, C—blue and green, Zn—yellow). (a) A top view of the computational structure; (c) A left view of the computational structure; The dashed purple lines indicate C-H···O hydrogen bonds **1**-**3**, where **1** = 2.15 Å (133.7°), **2** = 1.96 Å (145.4°), **3** = 1.90 Å (140.5°). All peripheral R1 and R2 group are replaced by CH_3 for simplicity.

Standard orientation for the optimized structure of **1a** ⊃ **G2** + Zn^{2+}

Center Number	Atomic Number	Atomic Type	Coordinates (Angstroms) X	Y	Z
1	6	0	-8.164908	-1.658407	-0.117726
2	6	0	-6.641133	-1.594524	-0.115842
3	6	0	-5.933548	-0.244660	-0.025764
4	6	0	-6.751810	1.040180	0.062354
5	6	0	-8.275586	0.976297	0.060470
6	6	0	-8.982158	-0.372470	-0.029534
7	7	0	-5.825074	-2.877342	-0.203818
8	7	0	-6.046207	2.386792	0.152212
9	6	0	-4.303412	-2.813548	-0.201937
10	8	0	-3.597806	-1.466936	-0.112079
11	6	0	-3.487351	-4.096367	-0.289916
12	6	0	-4.524545	2.450587	0.154094
13	8	0	-3.708484	1.167768	0.066117

14	6	0	-3.818940	3.797198	0.243954
15	6	0	-2.294107	3.861125	0.245840
16	6	0	-1.587535	5.209892	0.335843
17	6	0	-2.404785	6.495829	0.424033
18	6	0	-3.929617	6.431903	0.422148
19	6	0	-4.636146	5.082082	0.332074
20	6	0	-4.193923	-5.445134	-0.379919
21	6	0	-3.376717	-6.730017	-0.468039
22	6	0	-1.851885	-6.666090	-0.466153
23	6	0	-1.145312	-5.317325	-0.376150
24	6	0	-1.962518	-4.032440	-0.288030
25	6	0	0.376350	-5.253531	-0.374268
26	6	0	-0.065873	5.273686	0.337725
27	8	0	0.750186	3.990867	0.249746
28	7	0	0.639730	6.620300	0.427583
29	6	0	2.161394	6.684094	0.429465
30	7	0	1.192409	-6.536347	-0.462245
31	8	0	1.081953	-3.906917	-0.284408
32	6	0	2.714072	-6.472553	-0.460363
33	6	0	3.531320	-7.758491	-0.548553
34	6	0	5.056154	-7.694564	-0.546668
35	6	0	5.762681	-6.344744	-0.456594
36	6	0	4.945477	-5.059862	-0.368475
37	6	0	3.420643	-5.123789	-0.370360
38	6	0	2.978597	5.399209	0.341345
39	6	0	4.503432	5.463136	0.343231
40	6	0	5.210002	6.811903	0.433234
41	6	0	4.392754	8.097841	0.521425
42	6	0	2.867920	8.033914	0.519539
43	7	0	5.651080	-3.713249	-0.278616
44	7	0	5.319491	4.180317	0.255254
45	8	0	-9.091645	2.259116	0.148447
46	8	0	-8.870511	-3.005019	-0.207584
47	6	0	-10.613309	2.195322	0.146565

48	6	0	-10.392175	-3.068813	-0.209466
49	8	0	-6.157808	5.018288	0.330192
50	8	0	-1.699182	7.842443	0.513892
51	8	0	-5.715585	-5.508928	-0.381801
52	8	0	-1.035782	-7.949962	-0.554201
53	8	0	2.825717	-9.105104	-0.638414
54	8	0	7.284344	-6.280950	-0.454712
55	6	0	8.100403	-7.563769	-0.542690
56	6	0	3.641776	-10.387924	-0.726391
57	8	0	6.731665	6.875698	0.435116
58	8	0	2.051861	9.316732	0.607518
59	6	0	2.757464	10.663346	0.697376
60	6	0	7.437269	8.222310	0.524976
61	6	0	4.838367	-2.434514	-0.190919
62	6	0	5.540978	-1.092252	-0.101350
63	6	0	4.728310	0.185428	-0.013722
64	6	0	5.430921	1.527692	0.075847
65	6	0	4.616880	2.838055	0.165683
66	6	0	7.057358	-1.028679	-0.099475
67	6	0	6.947611	1.583883	0.077224
68	6	0	7.754159	0.300666	-0.010765
69	8	0	3.321988	-2.498086	-0.192794
70	8	0	3.100146	2.782917	0.164380
71	8	0	7.657795	2.922239	0.166518
72	8	0	7.871126	-2.307369	-0.187169
73	6	0	9.179812	2.977599	0.167829
74	6	0	9.392788	-2.243574	-0.185287
75	6	0	-6.421188	-6.855539	-0.471659
76	6	0	-1.741385	-9.296575	-0.644061
77	6	0	-2.515241	9.125262	0.601871
78	6	0	-6.973867	6.301108	0.418169
79	1	0	-4.834523	-0.198394	-0.024391
80	1	0	-10.081200	-0.418355	-0.030879
81	1	0	-6.293917	-3.772196	-0.263531

82	1	0	-6.588451	3.239258	0.210676
83	1	0	-1.704669	2.934560	0.182294
84	1	0	-4.519217	7.358366	0.485687
85	1	0	-3.886375	-7.702661	-0.532943
86	1	0	-1.452861	-3.059797	-0.223125
87	1	0	0.097486	7.472765	0.486047
88	1	0	0.723565	-7.431201	-0.521957
89	1	0	5.645754	-8.621027	-0.610207
90	1	0	2.831204	-4.197224	-0.306814
91	1	0	2.468939	4.426566	0.276441
92	1	0	4.902580	9.070397	0.586323
93	1	0	6.662200	-3.671069	-0.277381
94	1	0	6.330593	4.222916	0.256519
95	1	0	-11.028443	3.225610	0.216840
96	1	0	-10.962943	1.718561	-0.796421
97	1	0	-10.960392	1.595470	1.017473
98	1	0	-10.719443	-4.130226	-0.280658
99	1	0	-10.782815	-2.623088	0.732606
100	1	0	-10.786097	-2.500027	-1.081289
101	1	0	9.185305	-7.315713	-0.527662
102	1	0	7.857780	-8.217414	0.324864
103	1	0	7.854644	-8.095439	-1.489105
104	1	0	2.955039	-11.261882	-0.784346
105	1	0	4.281422	-10.358386	-1.636748
106	1	0	4.284949	-10.480978	0.177179
107	1	0	1.999845	11.476750	0.753545
108	1	0	3.395088	10.687357	1.609313
109	1	0	3.392820	10.810002	-0.204612
110	1	0	8.539160	8.065931	0.512651
111	1	0	7.142930	8.853443	-0.343243
112	1	0	7.145536	8.731445	1.470726
113	1	0	3.629284	0.139161	-0.015091
114	1	0	8.853317	0.343686	-0.009610
115	1	0	9.512984	4.037182	0.238890

116	1	0	9.570518	2.407118	1.039992
117	1	0	9.568007	2.529175	-0.773973
118	1	0	9.807922	-3.273863	-0.255561
119	1	0	9.739526	-1.644224	-1.056677
120	1	0	9.742765	-1.766252	0.757288
121	1	0	-7.523079	-6.699161	-0.459334
122	1	0	-6.129745	-7.364082	-1.417816
123	1	0	-6.126561	-7.487205	0.396076
124	1	0	-0.983766	-10.109980	-0.700227
125	1	0	-2.376114	-9.443177	0.258379
126	1	0	-2.379636	-9.320700	-1.555556
127	1	0	-1.828504	9.999220	0.659824
128	1	0	-3.157782	9.218314	-0.302149
129	1	0	-3.155520	9.095785	1.511784
130	1	0	-8.058768	6.053051	0.403141
131	1	0	-6.728348	6.832162	1.364991
132	1	0	-6.731002	6.955308	-0.448900
133	6	0	-0.365176	-0.088586	0.801905
134	6	0	-1.676870	-0.356525	3.074846
135	6	0	-1.672801	-0.498319	4.591225
136	7	0	-0.352968	-0.513969	5.351045
137	6	0	0.962797	-0.387824	4.594484
138	6	0	0.958726	-0.246030	3.078104
139	6	0	-0.361107	-0.230381	2.318284
140	6	0	0.942447	0.321148	-2.987414
141	6	0	0.938378	0.462942	-4.503792
142	7	0	-0.381455	0.478592	-5.263612
143	6	0	-1.697218	0.352448	-4.507053
144	6	0	-1.693149	0.210654	-2.990672
145	6	0	-0.369246	0.053209	-0.714475
146	6	0	-0.373316	0.195004	-2.230852
147	30	0	-0.348899	-0.655765	6.867424
148	30	0	-0.385524	0.620388	-6.779991
149	7	0	-0.389594	0.762182	-8.296370

150	7	0	-0.344829	-0.797559	8.383804
151	6	0	0.926169	0.888326	-9.052931
152	6	0	0.922099	1.030120	-10.569310
153	6	0	-0.397733	1.045769	-11.329129
154	6	0	-1.713496	0.919625	-10.572569
155	6	0	-1.709427	0.777831	-9.056190
156	6	0	-1.660593	-0.923703	9.140364
157	6	0	-1.656523	-1.065497	10.656743
158	6	0	-0.336690	-1.081146	11.416563
159	6	0	0.979074	-0.955002	10.660002
160	6	0	0.975004	-0.813208	9.143623
161	1	0	-2.630131	-0.345221	2.526061
162	1	0	-2.623122	-0.589427	5.137658
163	1	0	1.916058	-0.399127	5.143270
164	1	0	1.909047	-0.154921	2.531671
165	1	0	1.895708	0.309845	-2.438629
166	1	0	1.888699	0.554050	-5.050224
167	1	0	-2.650478	0.363751	-5.055839
168	1	0	-2.643470	0.119544	-2.444240
169	1	0	1.879430	0.877022	-8.504145
170	1	0	1.872421	1.121228	-11.115742
171	1	0	-0.400671	1.148182	-12.424348
172	1	0	-2.666757	0.930928	-11.121355
173	1	0	-2.659748	0.686722	-8.509757
174	1	0	-2.613854	-0.912399	8.591578
175	1	0	-2.606845	-1.156606	11.203176
176	1	0	-0.333751	-1.183559	12.511781
177	1	0	1.932334	-0.966306	11.208789
178	1	0	1.925325	-0.722099	8.597191

ESI-HRMS of [3]rotaxane and [2]rotaxane

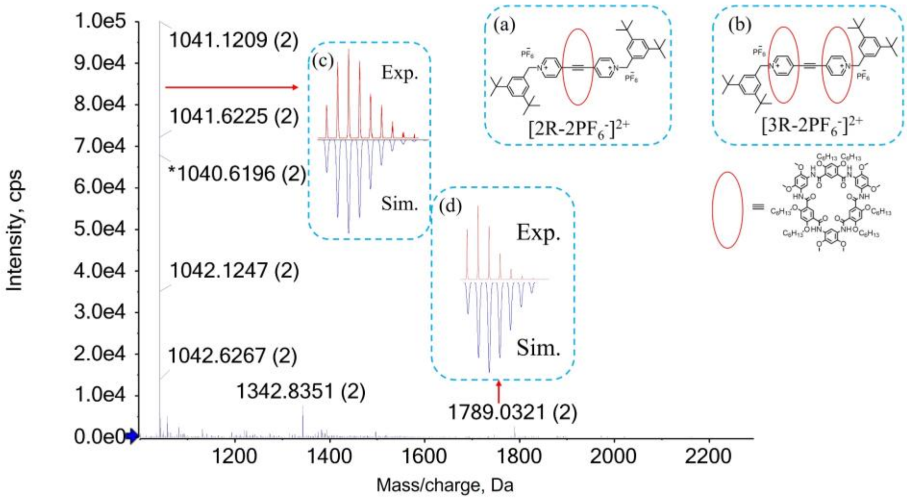

Figure S29 ESI-MS spectrum of [3]rotaxane and [2]rotaxane. (a) The structure of [2]rotaxane; (b) The structure of [2]rotaxane; (c) Experiment and simulation data of [2]rotaxane; (d) Experiment and simulation data of [3]rotaxane.

^{1}H NMR spectra of [3]rotaxane and [2]rotaxane

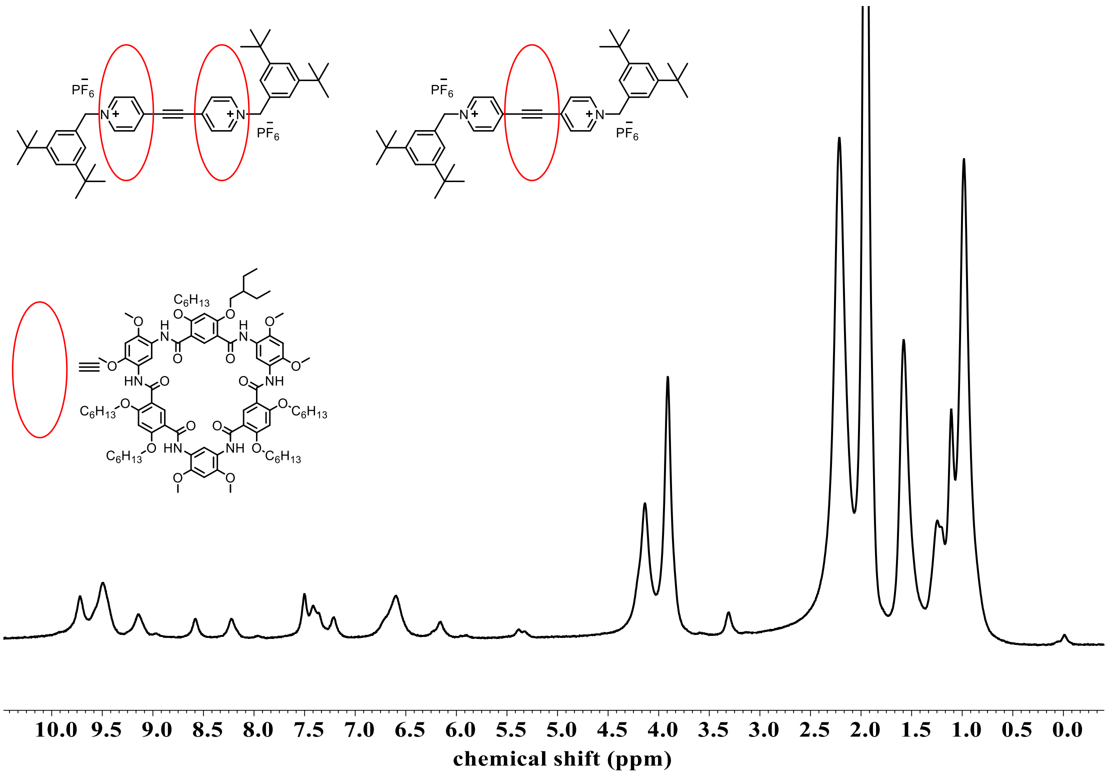

Figure S30 ^{1}H NMR spectra of [3]rotaxane and [2]rotaxane using **1a** and **G2** as the starting materials. (400 MHz, $CDCl_3/CD_3CN$, 1:1, v/v, 298K).

XPS of **1a** + **G2** + Zn^{2+} and **G2** + Zn^{2+}

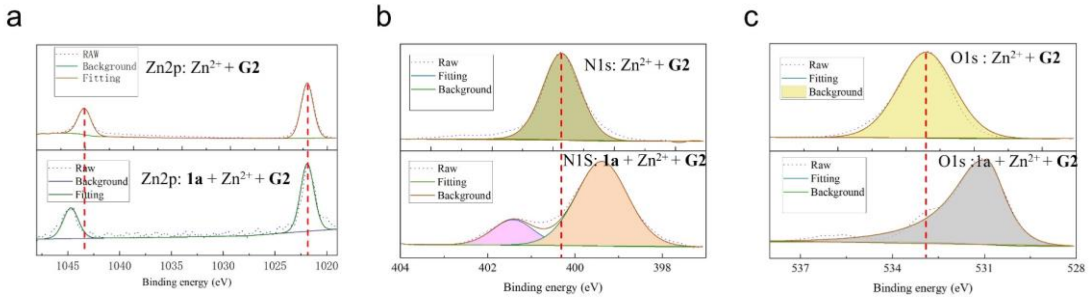

Figure S31 The XPS spectra of (a) Zn 2p spectra of Zn^{2+} + **G2** and **1a** + Zn^{2+} + **G2**; (b) N 1s spectra of Zn^{2+} + **G2** and **1a** + Zn^{2+} + **G2**; (c) O 1s spectra of Zn^{2+} + **G2** and **1a** + Zn^{2+} + **G2**.

Number of cycles DLS of **1a** + **G2·2H** + Zn^{2+} + **1-MEH**

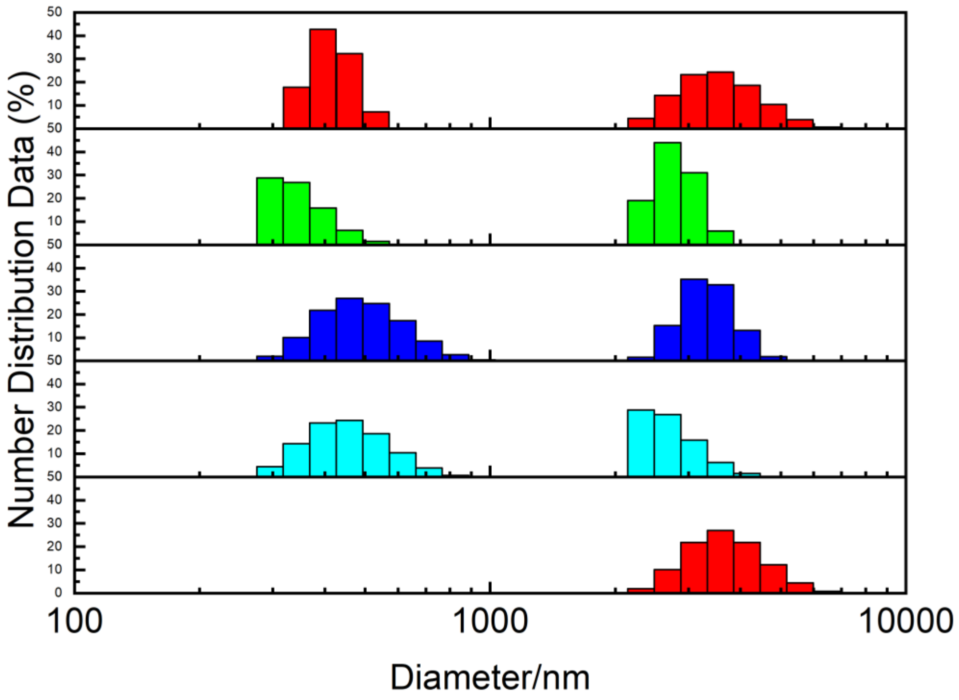

Figure S32 Number of cycles of DLS of **1a** + **G2** + Zn^{2+} + **1-MEH**. Solvent: $CHCl_3$: CH_3CN=1:1, *v/v*, 298 K.

TEM of **1a** + **G2** + Zn^{2+} + **1-MEH**

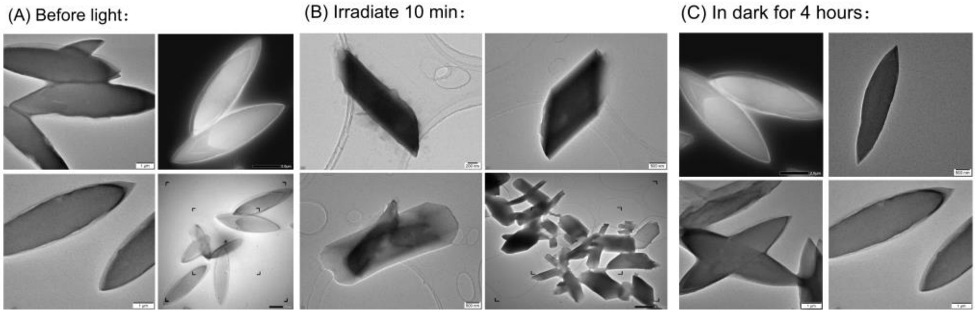

Figure S33 TEM of **1a** (2.0 eq.) + **G2** + Zn^{2+}(2.0 eq.) + **1-MEH** (2.4 eq.). (A) Before irradiation; (B) Irradiate 10 min; (C) In the dark for 240 min. [**G2**]= 25 μM. ($CHCl_3$: CH_3CN=1:1, *v/v*, 298K).

TEM of **1a** + **G2** + Zn^{2+} + **1-MEH**

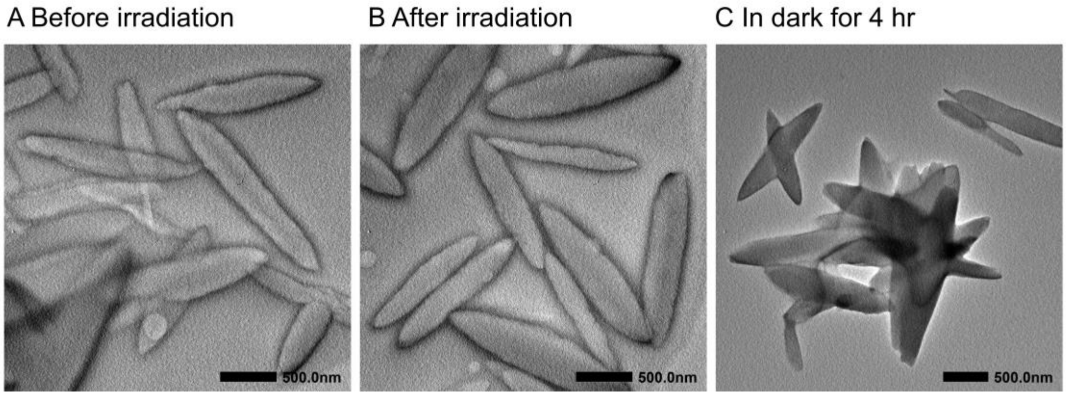

Figure S34 TEM of **G2** + Zn^{2+}(2.0 eq.) + **1-MEH** (2.4 eq.). (A) Before irradiation; (B) Irradiate 10 min; (C) In the dark for 240 min. [**G2**]= 25 μM. ($CHCl_3$: CH_3CN=1:1, *v/v*, 298K).

DFT calculation studies of **1a** and **G1•2H-G4•2H**

All side chains of **1a** are replaced by CH_3 for simplicity. The geometries of **G1•2H** ⊂**1a**, **G2•2H** ⊂**1a**, **G3•2H** ⊂**1a** and **G4•2H** ⊂**1a** were fully optimized at the B3LYP/ 6-311G (d,p) level of theory with the keyword UAHF method. The contained structures were confirmed without any imaginary frequency. All calculations were performed in Gaussian 09 program package.[5]

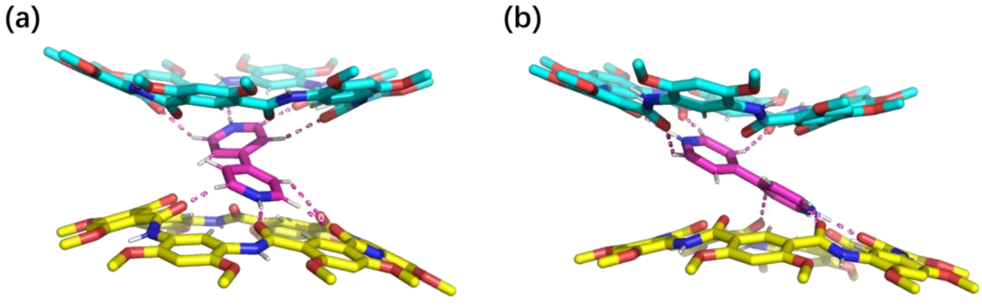

Figure S35 Optimized structure at the DFT/B3LYP/6-31G (d,p) level of complex **G1•2H** ⊂**1a**.(a) A front view of the computational structure; (b) A front view of the computational structure. All peripheral R^1 and R^2 group are replaced by CH_3 for simplicity.

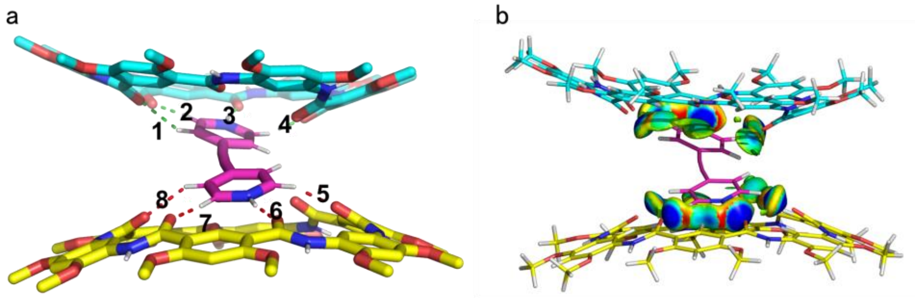

Figure S36 Optimized structure at the DFT/B3LYP/6-31G (d,p) level of complex **G2•2H** ⊂ **1a**. (a) A front view of the computational structure; (b) Independent gradient model (IGM) analysis of the computational structure (a) . All peripheral R^1 and R^2 group are replaced by CH_3 for simplicity.

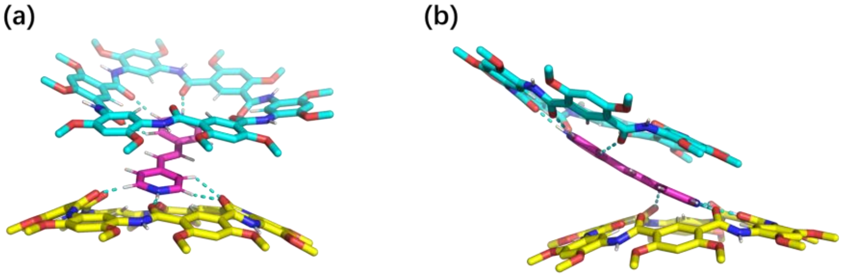

Figure S37 Optimized structure at the DFT/B3LYP/6-31G (d,p) level of complex **G3•2H** ⊂ **1a**. (a) A front view of the computational structure; (b) A front view of the computational structure. All peripheral R^1 and R^2 group are replaced by CH_3 for simplicity.

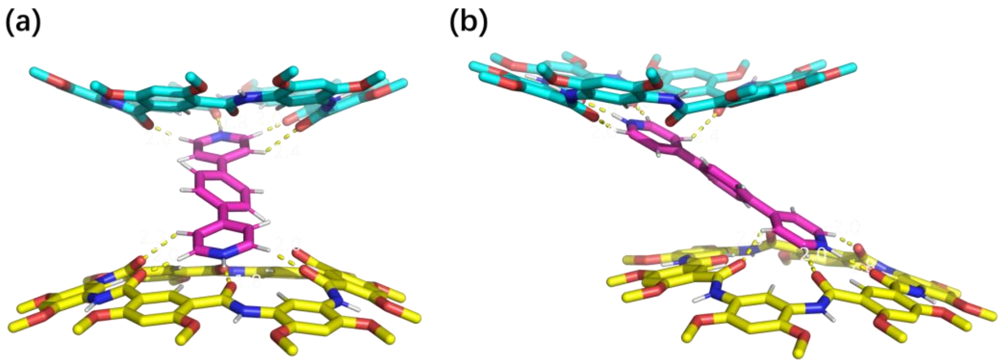

Figure S38 Optimized structure at the DFT/B3LYP/6-31G (d,p) level of complex **G4•2H** ⊂ **1a**. (a) A front view of the computational structure; (b) A front view of the computational structure. All peripheral R^1 and R^2 group are replaced by CH_3 for simplicity.

Standard orientation for the optimized structure of **1a ⊃ G1•2H**

Center Number	Atomic Number	Atomic Type	Coordinates (Angstroms) X	Y	Z
1	6	0	3.059159	7.828298	-4.413071
2	6	0	2.288384	6.512442	-4.387876
3	6	0	3.043287	5.185442	-4.376236
4	6	0	4.569035	5.176578	-4.389732
5	6	0	5.339725	6.492430	-4.414874
6	6	0	4.585245	7.817957	-4.426493
7	7	0	0.765612	6.522043	-4.374488
8	7	0	5.322174	3.853015	-4.378061
9	6	0	-0.004106	5.208077	-4.349377
10	8	0	0.749119	3.884518	-4.337759
11	6	0	-1.526962	5.217675	-4.335936
12	6	0	4.552542	2.539053	-4.353003
13	8	0	3.029685	2.548650	-4.339562
14	6	0	5.305696	1.215393	-4.341315
15	6	0	4.534429	-0.101270	-4.316199
16	6	0	5.288976	-1.426872	-4.304479
17	6	0	6.815131	-1.437112	-4.317973
18	6	0	7.586347	-0.120472	-4.343171
19	6	0	6.830933	1.205643	-4.354737
20	6	0	-2.281358	6.543205	-4.347608
21	6	0	-3.806595	6.552954	-4.334187
22	6	0	-4.577863	5.236292	-4.309071
23	6	0	-3.823401	3.910687	-4.297298
24	6	0	-2.298215	3.900915	-4.310803
25	6	0	-4.593033	2.596724	-4.272240
26	6	0	4.519343	-2.740834	-4.279421
27	8	0	2.996486	-2.731237	-4.265980
28	7	0	5.272483	-4.064397	-4.267750
29	6	0	4.502865	-5.378456	-4.242674
30	7	0	-6.115890	2.606322	-4.258799
31	8	0	-3.839809	1.273165	-4.260622
32	6	0	-6.885522	1.292360	-4.233741
33	6	0	-8.411608	1.302701	-4.220318
34	6	0	-9.182861	-0.014060	-4.195185
35	6	0	-8.427480	-1.340156	-4.183483
36	6	0	-6.902243	-1.349905	-4.196905

37	6	0	-6.131042	-0.033167	-4.222121
38	6	0	2.977664	-5.368587	-4.229187
39	6	0	2.206412	-6.685347	-4.204054
40	6	0	2.960907	-8.010972	-4.192417
41	6	0	4.486978	-8.021215	-4.205857
42	6	0	5.258245	-6.704552	-4.230973
43	7	0	-6.149103	-2.673468	-4.185234
44	7	0	0.683555	-6.675750	-4.190613
45	8	0	6.862597	6.482735	-4.428297
46	8	0	2.305935	9.151857	-4.424688
47	6	0	7.632214	7.796795	-4.453373
48	6	0	3.075567	10.465819	-4.449747
49	8	0	7.600599	2.519586	-4.379931
50	8	0	7.568286	-2.760772	-4.306284
51	8	0	-1.511792	7.857242	-4.372767
52	8	0	-6.101638	5.246380	-4.295559
53	8	0	-9.164832	2.626260	-4.231936
54	8	0	-9.197112	-2.654118	-4.158425
55	6	0	-10.719969	-2.644520	-4.144984
56	6	0	-10.687689	2.635857	-4.218495
57	8	0	2.191275	-9.324934	-4.167359
58	8	0	6.781102	-6.714150	-4.244414
59	6	0	7.534326	-8.037709	-4.232796
60	6	0	2.944414	-10.648496	-4.155688
61	6	0	-4.631504	-2.683744	-4.198622
62	6	0	-3.880381	-4.002389	-4.187047
63	6	0	-2.363630	-4.012074	-4.200433
64	6	0	-1.612493	-5.330816	-4.188840
65	6	0	-0.067582	-5.357007	-4.202206
66	6	0	-4.647391	-5.311860	-4.162058
67	6	0	-2.385891	-6.636520	-4.163829
68	6	0	-3.903957	-6.618629	-4.150541
69	8	0	-3.864545	-1.374295	-4.223694
70	8	0	0.706665	-4.051895	-4.227218
71	8	0	-1.642171	-7.959430	-4.152069
72	8	0	-6.165402	-5.302563	-4.148632
73	6	0	-2.419077	-9.269153	-4.127053
74	6	0	-6.935119	-6.616529	-4.123520
75	6	0	-2.265016	9.180801	-4.384385
76	6	0	-6.854792	6.570040	-4.307247
77	6	0	9.091142	-2.770370	-4.319725
78	6	0	9.123371	2.509985	-4.393319
79	1	0	2.487499	4.236331	-4.358096
80	1	0	5.141282	8.766902	-4.444622
81	1	0	0.265076	7.401580	-4.382216
82	1	0	6.334085	3.846532	-4.386995
83	1	0	3.434507	-0.094332	-4.306484
84	1	0	8.686291	-0.127192	-4.352838
85	1	0	-4.350593	7.509028	-4.342582
86	1	0	-1.754180	2.944961	-4.302342
87	1	0	6.284478	-4.070877	-4.276737
88	1	0	-6.616425	3.485860	-4.266526
89	1	0	-10.282838	-0.007320	-4.185382
90	1	0	-5.031069	-0.040083	-4.231753
91	1	0	2.433630	-4.412633	-4.237648
92	1	0	5.030840	-8.977315	-4.197492
93	1	0	-6.660699	-3.546551	-4.168550
94	1	0	0.172294	-7.548996	-4.173971
95	1	0	8.725258	7.586995	-4.460221
96	1	0	7.359038	8.368717	-5.368261
97	1	0	7.374943	8.394534	-3.550519
98	1	0	2.358035	11.316602	-4.455212

99	1	0	3.723182	10.533087	-3.547106
100	1	0	3.707630	10.507913	-5.365000
101	1	0	-11.097958	-3.691191	-4.127035
102	1	0	-11.079094	-2.105451	-3.239840
103	1	0	-11.095649	-2.130138	-5.057609
104	1	0	-11.052718	3.687250	-4.229852
105	1	0	-11.069437	2.101427	-5.117021
106	1	0	-11.053558	2.126124	-3.299200
107	1	0	8.629847	-7.841821	-4.245280
108	1	0	7.270018	-8.606251	-3.313130
109	1	0	7.253555	-8.632074	-5.131008
110	1	0	2.216400	-11.490089	-4.137483
111	1	0	3.575087	-10.723883	-5.069653
112	1	0	3.591906	-10.698733	-3.251812
113	1	0	-1.807927	-3.062966	-4.218520
114	1	0	-4.462412	-7.566088	-4.132411
115	1	0	-1.706320	-10.123931	-4.121530
116	1	0	-3.050804	-9.307636	-3.211424
117	1	0	-3.067609	-9.332884	-5.029247
118	1	0	-8.028148	-6.406826	-4.116655
119	1	0	-6.678121	-7.213696	-5.026964
120	1	0	-6.661651	-7.189012	-3.209142
121	1	0	-1.536865	10.022420	-4.402561
122	1	0	-2.911816	9.230966	-5.288669
123	1	0	-2.896330	9.256215	-3.470876
124	1	0	-7.950398	6.374149	-4.294710
125	1	0	-6.574305	7.163827	-3.408581
126	1	0	-6.590320	7.139104	-5.226433
127	1	0	9.456120	-3.821785	-4.308451
128	1	0	9.456627	-2.261187	-5.239477
129	1	0	9.473274	-2.235456	-3.421688
130	1	0	9.501396	3.556776	-4.411203
131	1	0	9.498674	1.996162	-3.480131
132	1	0	9.482840	1.970446	-5.297943
133	6	0	3.095646	8.061914	4.131109
134	6	0	2.324941	6.746159	4.156233
135	6	0	3.079871	5.419438	4.187688
136	6	0	4.605682	5.410974	4.194073
137	6	0	5.376387	6.726729	4.168949
138	6	0	4.621829	8.051954	4.137431
139	7	0	0.802072	6.755378	4.149876
140	7	0	5.358933	4.087695	4.225507
141	6	0	0.032440	5.441416	4.174934
142	8	0	0.785691	4.118137	4.206367
143	6	0	-1.490514	5.450633	4.168630
144	6	0	4.589216	2.773730	4.250618
145	8	0	3.066347	2.782950	4.244261
146	6	0	5.342452	1.450548	4.282034
147	6	0	4.571200	0.133787	4.307167
148	6	0	5.325758	-1.191437	4.338685
149	6	0	6.851941	-1.201397	4.345007
150	6	0	7.623193	0.115363	4.319874
151	6	0	6.867716	1.441078	4.288428
152	6	0	-2.244937	6.775883	4.137142
153	6	0	-3.770270	6.785252	4.130818
154	6	0	-4.541538	5.468589	4.155934
155	6	0	-3.786979	4.143365	4.187452
156	6	0	-2.261730	4.133993	4.193828
157	6	0	-4.556611	2.829403	4.212510
158	6	0	4.556126	-2.505399	4.363743
159	8	0	3.033257	-2.496179	4.357386
160	7	0	5.309377	-3.828678	4.395177

161	6	0	4.539744	-5.142641	4.420235
162	7	0	-6.079480	2.838622	4.206153
163	8	0	-3.803360	1.506123	4.243944
164	6	0	-6.849197	1.524657	4.231265
165	6	0	-8.375295	1.534620	4.224889
166	6	0	-9.146548	0.217860	4.250022
167	6	0	-8.391155	-1.107858	4.281522
168	6	0	-6.865891	-1.117327	4.287916
169	6	0	-6.094639	0.199433	4.262783
170	6	0	3.014495	-5.133269	4.413859
171	6	0	2.243279	-6.449909	4.439057
172	6	0	2.997787	-7.775156	4.470492
173	6	0	4.523936	-7.785097	4.476951
174	6	0	5.295137	-6.468359	4.451735
175	7	0	-6.112655	-2.440509	4.319331
176	7	0	0.720326	-6.440692	4.432753
177	8	0	6.899256	6.717509	4.175307
178	8	0	2.342395	9.385193	4.099676
179	6	0	7.668973	8.031475	4.150195
180	6	0	3.112112	10.699158	4.074564
181	8	0	7.637433	2.755044	4.263316
182	8	0	7.605177	-2.524579	4.376423
183	8	0	-1.475304	8.089845	4.112083
184	8	0	-6.065255	5.478400	4.149577
185	8	0	-9.128531	2.857802	4.193473
186	8	0	-9.160773	-2.421918	4.306598
187	6	0	-10.683605	-2.412578	4.300306
188	6	0	-10.651415	2.867120	4.187098
189	8	0	2.228070	-9.089121	4.495604
190	8	0	6.818020	-6.477676	4.458110
191	6	0	7.571257	-7.800858	4.489525
192	6	0	2.981321	-10.412401	4.527037
193	6	0	-4.594958	-2.450405	4.325689
194	6	0	-3.843776	-3.768789	4.356944
195	6	0	-2.327064	-3.778119	4.363273
196	6	0	-1.575844	-5.096383	4.394593
197	6	0	-0.030824	-5.122327	4.401362
198	6	0	-4.610749	-5.078141	4.381998
199	6	0	-2.349209	-6.402107	4.419468
200	6	0	-3.867358	-6.384693	4.413028
201	8	0	-3.828000	-1.140956	4.300617
202	8	0	0.743390	-3.817195	4.376486
203	8	0	-1.605429	-7.724756	4.450908
204	8	0	-6.128992	-5.069250	4.375649
205	6	0	-2.382401	-9.034404	4.475824
206	6	0	-6.898574	-6.383190	4.400791
207	6	0	-2.228555	9.413125	4.080650
208	6	0	-6.818491	6.801582	4.118162
209	6	0	9.128061	-2.533896	4.382798
210	6	0	9.160251	2.745801	4.269591
211	1	0	2.524219	4.470354	4.205858
212	1	0	5.177866	9.000898	4.119303
213	1	0	0.301524	7.634697	4.128944
214	1	0	6.370927	4.081531	4.229707
215	1	0	3.471298	0.140419	4.302595
216	1	0	8.723217	0.108855	4.324458
217	1	0	-4.314300	7.740997	4.108054
218	1	0	-1.717652	3.178131	4.216475
219	1	0	6.321370	-3.834842	4.399377
220	1	0	-6.580113	3.717938	4.185274
221	1	0	-10.246571	0.224369	4.245438
222	1	0	-4.994637	0.192707	4.267319

223	1	0	2.470418	-4.177407	4.391212
224	1	0	5.067793	-8.740988	4.499620
225	1	0	-6.624265	-3.313495	4.335998
226	1	0	0.209064	-7.313938	4.449396
227	1	0	8.761964	7.822032	4.159089
228	1	0	7.407458	8.596352	3.227493
229	1	0	7.399859	8.635970	5.045127
230	1	0	2.394534	11.549678	4.053393
231	1	0	3.747930	10.773446	4.984989
232	1	0	3.755901	10.734511	3.167421
233	1	0	-11.061646	-3.459244	4.321182
234	1	0	-11.054489	-1.866817	5.196529
235	1	0	-11.047540	-1.905306	3.378866
236	1	0	-11.016492	3.918276	4.163045
237	1	0	-11.021452	2.325788	3.287806
238	1	0	-11.029129	2.364265	5.105438
239	1	0	8.666924	-7.604829	4.489702
240	1	0	7.295238	-8.362500	5.409957
241	1	0	7.302382	-8.402079	3.592351
242	1	0	2.253256	-11.254043	4.542150
243	1	0	3.623837	-10.494476	3.621940
244	1	0	3.617101	-10.455546	5.439591
245	1	0	-1.771375	-2.828914	4.345168
246	1	0	-4.425864	-7.332175	4.431076
247	1	0	-1.669598	-9.888919	4.497052
248	1	0	-3.025937	-9.066150	5.383395
249	1	0	-3.019172	-9.105275	3.565779
250	1	0	-7.991700	-6.173773	4.391867
251	1	0	-6.629818	-6.987117	3.505321
252	1	0	-6.636888	-6.948751	5.322971
253	1	0	-1.500491	10.254767	4.065537
254	1	0	-2.863779	9.456173	3.167658
255	1	0	-2.871728	9.495325	4.985274
256	1	0	-7.914074	6.605556	4.117932
257	1	0	-6.549849	7.402248	5.015872
258	1	0	-6.542238	7.363846	3.198140
259	1	0	9.493137	-3.585053	4.406852
260	1	0	9.505441	-2.031570	3.464084
261	1	0	9.498448	-1.992062	5.281737
262	1	0	9.538292	3.792467	4.248714
263	1	0	9.523857	2.238971	5.191474
264	1	0	9.531527	2.199452	3.373769
265	6	0	-1.030946	-0.893279	-3.121330
266	7	0	0.289100	-0.883390	-3.880910
267	6	0	1.604071	-0.770633	-3.120989
268	6	0	1.599117	-0.667641	-1.601475
269	6	0	0.279057	-0.677432	-0.841912
270	6	0	-1.035900	-0.790287	-1.601816
271	6	0	0.274103	-0.574440	0.677601
272	6	0	1.589210	-0.461657	1.437552
273	6	0	1.584205	-0.358687	2.956983
274	7	0	0.264210	-0.368553	3.716646
275	6	0	-1.050897	-0.481336	2.956695
276	6	0	-1.045943	-0.584328	1.437181
277	1	0	-1.980779	-0.974767	-3.670187
278	1	0	0.292478	-0.954065	-4.923460
279	1	0	2.557505	-0.763511	-3.669677
280	1	0	2.548950	-0.586152	-1.052618
281	1	0	-1.989334	-0.797408	-1.053127
282	1	0	2.542559	-0.454538	0.888916
283	1	0	2.533953	-0.277202	3.505893
284	1	0	0.260747	-0.297881	4.759249

285	1	0	-2.004246	-0.488455	3.505330
286	1	0	-1.995640	-0.665791	0.888354

Standard orientation for the optimized structure of **1a** ⊃ **G2•2H**

Center Number	Atomic Number		Coordinates (Angstroms) X	Y	Z
1	6	0	7.491592	7.156007	1.611296
2	6	0	6.997498	5.890261	1.243947
3	6	0	5.670689	5.565938	1.535799
4	6	0	4.833680	6.462120	2.203417
5	6	0	5.336822	7.727039	2.565682
6	6	0	6.658851	8.069199	2.265197
7	7	0	7.909588	4.977952	0.664166
8	7	0	3.522005	6.137159	2.611119
9	6	0	7.621513	3.734064	0.186806
10	8	0	6.465552	3.319768	0.049509
11	6	0	8.764196	2.797244	-0.109891
12	6	0	2.834463	4.989597	2.320186
13	8	0	3.228938	4.163886	1.491569
14	6	0	1.591989	4.692347	3.116478
15	6	0	1.229206	3.347529	3.147937
16	6	0	0.125665	2.836870	3.829314
17	6	0	-0.683317	3.767820	4.517171
18	6	0	-0.352661	5.130297	4.519089
19	6	0	0.780197	5.592734	3.837427
20	6	0	10.138249	3.090796	-0.238797
21	6	0	11.056692	2.060704	-0.484654
22	6	0	10.636360	0.727447	-0.577216
23	6	0	9.266684	0.397391	-0.437775
24	6	0	8.393389	1.460813	-0.224421
25	6	0	8.628427	-0.965655	-0.425524
26	6	0	-0.014782	1.334840	3.744534
27	8	0	0.849186	0.686240	3.152731
28	7	0	-1.110495	0.756294	4.328658
29	6	0	-1.387994	-0.623172	4.428214
30	7	0	9.450071	-2.047794	-0.429901
31	8	0	7.389916	-1.070267	-0.366996
32	6	0	9.115629	-3.408302	-0.240616
33	6	0	10.174468	-4.335051	-0.329768
34	6	0	9.944346	-5.690186	-0.083494
35	6	0	8.661739	-6.134335	0.250377
36	6	0	7.594001	-5.219737	0.331311
37	6	0	7.836484	-3.865303	0.083645
38	6	0	-0.622201	-1.624801	3.822411
39	6	0	-0.932586	-2.977099	3.999662
40	6	0	-2.035673	-3.333843	4.796091
41	6	0	-2.821671	-2.341729	5.390028
42	6	0	-2.503798	-0.994506	5.207421
43	7	0	6.342381	-5.721009	0.747801
44	7	0	-0.129457	-4.023310	3.496756
45	8	0	4.457583	8.546157	3.226390
46	8	0	8.807243	7.392236	1.300811
47	6	0	4.917880	9.809762	3.681600
48	6	0	9.385869	8.627585	1.695318
49	8	0	1.148085	6.905744	3.826056
50	8	0	-1.777683	3.285775	5.172232
51	8	0	10.508794	4.393759	-0.120956
52	8	0	11.507701	-0.293181	-0.804470
53	8	0	11.399018	-3.807135	-0.649584

54	8	0	8.346584	-7.436995	0.533594
55	6	0	9.381041	-8.410056	0.500809
56	6	0	12.516658	-4.680901	-0.727719
57	8	0	-2.247293	-4.681533	4.949205
58	8	0	-3.216162	0.045256	5.753634
59	6	0	-4.326866	-0.256048	6.582694
60	6	0	-3.329756	-5.107644	5.761038
61	6	0	5.136590	-5.085295	0.647608
62	6	0	3.966951	-5.666152	1.395771
63	6	0	2.890255	-4.796136	1.576742
64	6	0	1.750150	-5.088587	2.322931
65	6	0	0.779582	-3.939600	2.476986
66	6	0	3.863277	-6.944065	1.987352
67	6	0	1.677448	-6.376860	2.897004
68	6	0	2.720202	-7.294961	2.715665
69	8	0	5.000892	-4.025931	0.028379
70	8	0	0.885498	-2.949361	1.751736
71	8	0	0.560596	-6.679643	3.617218
72	8	0	4.908134	-7.803662	1.811792
73	6	0	0.438040	-7.952946	4.243839
74	6	0	4.875691	-9.094319	2.415029
75	6	0	11.883253	4.758075	-0.238052
76	6	0	12.903567	-0.030664	-0.937443
77	6	0	-2.628394	4.173735	5.890517
78	6	0	0.357085	7.868066	4.517210
79	1	0	5.283542	4.607282	1.245350
80	1	0	7.041259	9.040350	2.547058
81	1	0	8.881047	5.267297	0.663466
82	1	0	3.075065	6.802981	3.230834
83	1	0	1.854917	2.637969	2.624979
84	1	0	-0.976968	5.830870	5.054265
85	1	0	12.104742	2.296537	-0.597893
86	1	0	7.344798	1.236694	-0.114851
87	1	0	-1.748686	1.366901	4.825772
88	1	0	10.442866	-1.864022	-0.533103
89	1	0	10.760557	-6.395808	-0.145970
90	1	0	7.025932	-3.162810	0.144431
91	1	0	0.226279	-1.345190	3.222469
92	1	0	-3.672332	-2.618170	5.996773
93	1	0	6.348344	-6.647326	1.159227
94	1	0	-0.250707	-4.925863	3.940359
95	1	0	4.072317	10.272060	4.193723
96	1	0	5.753421	9.702486	4.385103
97	1	0	5.225739	10.449192	2.844347
98	1	0	10.426913	8.590718	1.369853
99	1	0	8.886904	9.476141	1.210243
100	1	0	9.350540	8.757022	2.784568
101	1	0	8.908344	-9.360109	0.755225
102	1	0	9.826524	-8.485883	-0.499124
103	1	0	10.165582	-8.186897	1.235062
104	1	0	13.371270	-4.054886	-0.989637
105	1	0	12.708686	-5.172164	0.234514
106	1	0	12.375228	-5.441374	-1.505826
107	1	0	-4.728986	0.704395	6.910343
108	1	0	-5.103176	-0.802607	6.031499
109	1	0	-4.025042	-0.840202	7.461626
110	1	0	-3.309836	-6.198866	5.742165
111	1	0	-3.217785	-4.761152	6.796840
112	1	0	-4.291154	-4.759110	5.362235
113	1	0	2.956286	-3.810350	1.138200
114	1	0	2.645329	-8.281144	3.150143
115	1	0	-0.522562	-7.933015	4.759398

116	1	0	0.437207	-8.759560	3.501466
117	1	0	1.241227	-8.117532	4.971676
118	1	0	5.821211	-9.566424	2.146564
119	1	0	4.798796	-9.020520	3.506046
120	1	0	4.044987	-9.692376	2.022828
121	1	0	11.910889	5.839316	-0.101909
122	1	0	12.487880	4.274522	0.537801
123	1	0	12.273827	4.504374	-1.229999
124	1	0	13.367344	-1.003689	-1.099897
125	1	0	13.101042	0.617806	-1.798322
126	1	0	13.308917	0.422400	-0.025642
127	1	0	-3.413340	3.546615	6.314373
128	1	0	-2.084961	4.675591	6.699621
129	1	0	-3.076019	4.919485	5.223214
130	1	0	0.850113	8.826514	4.351450
131	1	0	-0.660594	7.906020	4.111664
132	1	0	0.321122	7.655558	5.592350
133	6	0	3.881522	-1.229613	-0.419046
134	7	0	4.711133	-0.171785	-0.268711
135	6	0	4.262114	1.082056	-0.032546
136	6	0	2.903199	1.320211	0.059758
137	6	0	2.005121	0.246193	-0.095797
138	6	0	2.515645	-1.046652	-0.340021
139	6	0	0.599714	0.407122	-0.013662
140	6	0	-0.613022	0.406313	0.018525
141	6	0	-2.017985	0.243094	0.102651
142	6	0	-2.918288	1.314732	-0.057176
143	6	0	-4.276670	1.074670	0.037476
144	7	0	-4.722927	-0.178702	0.281074
145	6	0	-3.891401	-1.234078	0.436761
146	6	0	-2.525956	-1.049430	0.354383
147	1	0	4.345109	-2.199152	-0.559540
148	1	0	5.728532	-0.354596	-0.316599
149	1	0	5.007554	1.864759	0.073206
150	1	0	2.566797	2.326270	0.282440
151	1	0	1.840454	-1.887853	-0.436547
152	1	0	-2.584160	2.320500	-0.284363
153	1	0	-5.024031	1.855035	-0.071616
154	1	0	-5.739701	-0.365328	0.329061
155	1	0	-4.353993	-2.202948	0.583236
156	1	0	-1.848996	-1.888813	0.454712
157	6	0	-5.411133	7.709928	-2.543501
158	6	0	-4.897804	6.448146	-2.184383
159	6	0	-5.728063	5.543095	-1.520371
160	6	0	-7.057864	5.855639	-1.229107
161	6	0	-7.562172	7.118162	-1.593532
162	6	0	-6.736319	8.040206	-2.243764
163	7	0	-3.582874	6.135911	-2.591355
164	7	0	-7.962985	4.933960	-0.653231
165	6	0	-2.886056	4.992731	-2.305314
166	8	0	-3.275427	4.158591	-1.482663
167	6	0	-1.638408	4.711712	-3.099425
168	6	0	-7.664324	3.692485	-0.176174
169	8	0	-6.504827	3.289444	-0.035100
170	6	0	-8.798816	2.743945	0.114891
171	6	0	-8.414843	1.411267	0.229315
172	6	0	-9.278140	0.338470	0.436195
173	6	0	-10.651840	0.654131	0.569085
174	6	0	-11.085402	1.983185	0.477047
175	6	0	-10.176420	3.023166	0.237606
176	6	0	-0.834152	5.623350	-3.814737
177	6	0	0.305090	5.175466	-4.495442

178	6	0	0.650260	3.816599	-4.497717
179	6	0	-0.150370	2.874643	-3.815105
180	6	0	-1.261241	3.370951	-3.135084
181	6	0	0.006266	1.373977	-3.734794
182	6	0	-8.625952	-1.017887	0.424048
183	8	0	-7.386003	-1.109736	0.374566
184	7	0	-9.436249	-2.108430	0.417622
185	6	0	-9.086292	-3.464557	0.224439
186	7	0	1.110257	0.809428	-4.316973
187	8	0	-0.852474	0.714072	-3.147883
188	6	0	1.403544	-0.566559	-4.419445
189	6	0	2.526970	-0.923176	-5.194606
190	6	0	2.860810	-2.266204	-5.379512
191	6	0	2.083361	-3.268818	-4.791855
192	6	0	0.972757	-2.926638	-3.999614
193	6	0	0.646378	-1.578456	-3.820041
194	6	0	-7.802546	-3.905702	-0.103991
195	6	0	-7.544926	-5.256580	-0.356061
196	6	0	-8.602113	-6.183593	-0.275492
197	6	0	-9.889107	-5.755195	0.061816
198	6	0	-10.134306	-4.403480	0.312489
199	7	0	0.178383	-3.982672	-3.503267
200	7	0	-6.288391	-5.743420	-0.774892
201	8	0	-8.880157	7.342393	-1.284116
202	8	0	-4.537860	8.538352	-3.200541
203	6	0	-9.468586	8.574043	-1.675726
204	6	0	-5.008250	9.799184	-3.653092
205	8	0	-10.559770	4.322441	0.120323
206	8	0	-11.513714	-0.375946	0.789742
207	8	0	-1.215485	6.932488	-3.799048
208	8	0	1.751124	3.348644	-5.152036
209	8	0	3.230058	0.126115	-5.734589
210	8	0	2.310778	-4.613551	-4.947676
211	6	0	3.403924	-5.025123	-5.752666
212	6	0	4.349126	-0.160200	-6.557664
213	8	0	-8.272598	-7.481945	-0.562221
214	8	0	-11.364363	-3.890506	0.635549
215	6	0	-12.471890	-4.777202	0.711751
216	6	0	-9.295617	-8.466993	-0.528541
217	6	0	-0.733409	-3.911510	-2.484926
218	6	0	-1.696619	-5.067861	-2.340674
219	6	0	-2.842378	-4.786023	-1.599409
220	6	0	-3.913804	-5.664233	-1.426457
221	6	0	-5.090157	-5.092691	-0.681630
222	6	0	-1.612265	-6.353283	-2.919463
223	6	0	-3.798187	-6.939369	-2.021664
224	6	0	-2.649383	-7.279383	-2.746283
225	8	0	-0.847899	-2.926488	-1.753853
226	8	0	-4.964788	-4.027466	-0.070372
227	8	0	-4.837400	-7.807327	-1.853583
228	8	0	-0.490028	-6.645250	-3.635737
229	6	0	-4.793716	-9.094787	-2.462842
230	6	0	-0.355464	-7.915473	-4.266122
231	6	0	-0.433823	7.905469	-4.485864
232	6	0	2.594433	4.248319	-5.864391
233	6	0	-12.912919	-0.128072	0.915843
234	6	0	-11.938531	4.672315	0.230872
235	1	0	-5.333258	4.586843	-1.232341
236	1	0	-7.126460	9.008979	-2.523224
237	1	0	-3.140567	6.808801	-3.206761
238	1	0	-8.937154	5.214066	-0.654997
239	1	0	-7.363343	1.198815	0.124819

240	1	0	-12.136351	2.207961	0.585582
241	1	0	0.923202	5.884617	-5.026467
242	1	0	-1.880930	2.652673	-2.616893
243	1	0	-10.431413	-1.935623	0.516827
244	1	0	1.743469	1.428577	-4.809864
245	1	0	3.717221	-2.531366	-5.983192
246	1	0	-0.207762	-1.309919	-3.223063
247	1	0	-7.000131	-3.193914	-0.164803
248	1	0	-10.697096	-6.470316	0.123612
249	1	0	0.308235	-4.882045	-3.950882
250	1	0	-6.284444	-6.670630	-1.184406
251	1	0	-10.509688	8.527538	-1.351687
252	1	0	-8.977303	9.425272	-1.187505
253	1	0	-9.432946	8.706983	-2.764544
254	1	0	-4.165888	10.270117	-4.162612
255	1	0	-5.841724	9.686590	-4.358208
256	1	0	-5.322929	10.433680	-2.814621
257	1	0	3.396909	-6.116534	-5.735946
258	1	0	3.295128	-4.678031	-6.788622
259	1	0	4.358180	-4.665855	-5.346352
260	1	0	4.742263	0.805684	-6.880190
261	1	0	5.128199	-0.699469	-6.003202
262	1	0	4.059417	-0.745270	-7.440073
263	1	0	-13.333294	-4.161888	0.976748
264	1	0	-12.659276	-5.267314	-0.251983
265	1	0	-12.320978	-5.538684	1.487084
266	1	0	-8.812505	-9.411152	-0.785321
267	1	0	-9.737983	-8.549286	0.472258
268	1	0	-10.084307	-8.251952	-1.260771
269	1	0	-2.918030	-3.802269	-1.157848
270	1	0	-2.565511	-8.263411	-3.184032
271	1	0	-5.737297	-9.574436	-2.200981
272	1	0	-4.712404	-9.015117	-3.553116
273	1	0	-3.960833	-9.689278	-2.069818
274	1	0	0.607452	-7.887052	-4.776947
275	1	0	-0.352570	-8.724620	-3.526510
276	1	0	-1.153954	-8.083187	-4.998403
277	1	0	-0.937064	8.858095	-4.317256
278	1	0	0.582966	7.952675	-4.079058
279	1	0	-0.394505	7.697165	-5.561707
280	1	0	3.386834	3.631093	-6.288891
281	1	0	2.047879	4.747982	-6.672755
282	1	0	3.032697	4.995799	-5.192805
283	1	0	-13.367544	-1.106212	1.073370
284	1	0	-13.121643	0.516156	1.777254
285	1	0	-13.318021	0.323132	0.003010
286	1	0	-11.976588	5.753499	0.096829
287	1	0	-12.533976	4.184188	-0.549205
288	1	0	-12.331717	4.412472	1.220194

Standard orientation for the optimized structure of **1a ⊃ G3•2H**

Center Number	Atomic Number	Atomic Type	Coordinates (Angstroms) X	Y	Z
1	6	0	1.015319	-6.413326	2.618939
2	6	0	1.355188	-5.159225	2.076460
3	6	0	2.601050	-5.002084	1.461004
4	6	0	3.523788	-6.059592	1.406490
5	6	0	3.163333	-7.306094	1.945156
6	6	0	1.913126	-7.478254	2.541868

7	7	0	0.444722	-4.097918	2.245856
8	7	0	4.846947	-5.940623	0.902374
9	6	0	0.587437	-2.817979	1.773053
10	8	0	1.455783	-2.509107	0.954349
11	6	0	-0.324433	-1.750058	2.347920
12	6	0	5.422435	-4.834786	0.366360
13	8	0	4.771566	-3.819147	0.088917
14	6	0	6.910888	-4.819941	0.153728
15	6	0	7.419658	-3.560576	-0.141428
16	6	0	8.754720	-3.244636	-0.347566
17	6	0	9.669753	-4.319372	-0.265390
18	6	0	9.209929	-5.612936	0.037984
19	6	0	7.846515	-5.866806	0.258862
20	6	0	-1.424444	-1.909486	3.221353
21	6	0	-2.101671	-0.793198	3.721140
22	6	0	-1.696853	0.503962	3.386330
23	6	0	-0.600649	0.712749	2.515598
24	6	0	0.031595	-0.435042	2.021650
25	6	0	0.042408	2.020803	2.089540
26	6	0	8.996109	-1.775686	-0.600287
27	8	0	8.038153	-0.983253	-0.594608
28	7	0	10.267198	-1.378208	-0.831168
29	6	0	10.715465	-0.056708	-1.033897
30	7	0	-0.402711	3.180418	2.668590
31	8	0	0.968693	2.004984	1.277751
32	6	0	0.199963	4.450004	2.572721
33	6	0	-0.405159	5.514781	3.270075
34	6	0	0.169419	6.785862	3.250758
35	6	0	1.353492	7.010588	2.547556
36	6	0	1.974497	5.961346	1.850272
37	6	0	1.378179	4.688251	1.861163
38	6	0	9.863459	1.046221	-1.022252
39	6	0	10.355457	2.340285	-1.205950
40	6	0	11.735590	2.524780	-1.419541
41	6	0	12.595523	1.425399	-1.442975
42	6	0	12.092719	0.137692	-1.251178
43	7	0	3.217673	6.259408	1.228075
44	7	0	9.543491	3.493508	-1.140736
45	8	0	4.118324	-8.285583	1.853940
46	8	0	-0.217995	-6.491387	3.216854
47	6	0	3.834869	-9.559968	2.411122
48	6	0	-0.599838	-7.720302	3.815889
49	8	0	7.360071	-7.099706	0.562187
50	8	0	10.984061	-4.042157	-0.483817
51	8	0	-1.799820	-3.180975	3.555128
52	8	0	-2.345014	1.599739	3.885717
53	8	0	-1.559458	5.208617	3.952953
54	8	0	2.005406	8.217679	2.494192
55	6	0	1.456596	9.314137	3.206952
56	6	0	-2.187833	6.236273	4.700641
57	8	0	12.143065	3.823072	-1.579801
58	8	0	12.860901	-0.998542	-1.254956
59	6	0	14.261735	-0.871704	-1.458309
60	6	0	13.526415	4.079507	-1.781068
61	6	0	4.000768	5.413726	0.495730
62	6	0	5.412498	5.848806	0.151512
63	6	0	6.199000	4.799479	-0.323310
64	6	0	7.546774	4.874825	-0.667731
65	6	0	8.191721	3.538969	-0.964133
66	6	0	6.037087	7.107308	0.273898
67	6	0	8.131692	6.167988	-0.564230
68	6	0	7.377063	7.258147	-0.103759

69	8	0	3.616935	4.295175	0.146882
70	8	0	7.493824	2.520903	-0.975045
71	8	0	9.442588	6.320111	-0.908902
72	8	0	5.288907	8.145734	0.743264
73	6	0	10.073738	7.593806	-0.784780
74	6	0	5.881297	9.433554	0.901197
75	6	0	-2.875822	-3.392322	4.461191
76	6	0	-3.422892	1.434627	4.798805
77	6	0	11.963203	-5.076184	-0.392159
78	6	0	8.249516	-8.207700	0.696484
79	1	0	2.855899	-4.042625	1.046760
80	1	0	1.644760	-8.439256	2.959293
81	1	0	-0.339322	-4.285941	2.857836
82	1	0	5.447273	-6.744423	1.049960
83	1	0	6.724815	-2.747222	-0.208753
84	1	0	9.918035	-6.426377	0.101193
85	1	0	-2.944462	-0.933769	4.383500
86	1	0	0.877879	-0.294786	1.361207
87	1	0	10.985092	-2.094315	-0.826663
88	1	0	-1.193421	3.119100	3.296989
89	1	0	-0.299870	7.598843	3.788563
90	1	0	1.834916	3.877526	1.321814
91	1	0	8.809074	0.896913	-0.883796
92	1	0	13.653782	1.571780	-1.609529
93	1	0	3.598439	7.176758	1.426512
94	1	0	10.036691	4.378024	-1.153684
95	1	0	4.721025	-10.172673	2.236123
96	1	0	3.648303	-9.492500	3.490656
97	1	0	2.971293	-10.027405	1.920517
98	1	0	-1.595170	-7.559561	4.234871
99	1	0	-0.647838	-8.529685	3.075711
100	1	0	0.089250	-8.005693	4.621182
101	1	0	2.128770	10.156872	3.034366
102	1	0	0.455320	9.571554	2.837061
103	1	0	1.403613	9.107916	4.284005
104	1	0	-3.068484	5.784644	5.162309
105	1	0	-1.526668	6.622948	5.487158
106	1	0	-2.506120	7.065670	4.055268
107	1	0	14.668576	-1.883746	-1.419813
108	1	0	14.484521	-0.432179	-2.438811
109	1	0	14.727280	-0.264501	-0.671446
110	1	0	13.624417	5.161989	-1.880318
111	1	0	14.125648	3.738188	-0.927174
112	1	0	13.892523	3.601048	-2.698229
113	1	0	5.642911	3.877061	-0.339301
114	1	0	7.842229	8.231469	-0.031106
115	1	0	11.105489	7.442543	-1.103029
116	1	0	9.595861	8.334301	-1.436099
117	1	0	10.057409	7.943659	0.253871
118	1	0	5.094753	10.073541	1.302217
119	1	0	6.721506	9.399769	1.604594
120	1	0	6.218988	9.831535	-0.062735
121	1	0	-2.959859	-4.474293	4.570869
122	1	0	-2.669393	-2.940808	5.438676
123	1	0	-3.814480	-2.991907	4.058665
124	1	0	-3.749396	2.443561	5.054140
125	1	0	-4.253651	0.888689	4.335095
126	1	0	-3.099775	0.913454	5.707756
127	1	0	12.919810	-4.593396	-0.593331
128	1	0	11.979330	-5.517965	0.610812
129	1	0	11.783056	-5.855898	-1.141089
130	1	0	7.620462	-9.060324	0.953521

131	1	0	8.770407	-8.410026	-0.246383
132	1	0	8.978806	-8.035824	1.496501
133	6	0	-4.421556	-7.940159	0.334652
134	6	0	-4.100365	-6.572084	0.354496
135	6	0	-3.819400	-5.928146	-0.860118
136	6	0	-3.910439	-6.598049	-2.086895
137	6	0	-4.233578	-7.966393	-2.083852
138	6	0	-4.474933	-8.629401	-0.877562
139	7	0	-4.151169	-5.903933	1.606018
140	7	0	-3.778916	-5.961070	-3.347609
141	6	0	-3.947745	-4.575127	1.839373
142	8	0	-3.513227	-3.817911	0.976669
143	6	0	-4.342978	-3.987592	3.175277
144	6	0	-3.550319	-4.631126	-3.584685
145	8	0	-3.184704	-3.838293	-2.715388
146	6	0	-3.818031	-4.089628	-4.961164
147	6	0	-3.854819	-2.698391	-5.015879
148	6	0	-4.172749	-1.947428	-6.144421
149	6	0	-4.432603	-2.674897	-7.332527
150	6	0	-4.395103	-4.077013	-7.327590
151	6	0	-4.109445	-4.785273	-6.154446
152	6	0	-4.812089	-4.639876	4.341753
153	6	0	-5.189798	-3.890019	5.462948
154	6	0	-5.133704	-2.487912	5.450077
155	6	0	-4.681139	-1.799915	4.301159
156	6	0	-4.292464	-2.589655	3.219653
157	6	0	-4.615293	-0.305548	4.043603
158	6	0	-4.248462	-0.456562	-5.886978
159	8	0	-4.062250	-0.034490	-4.746331
160	7	0	-4.557048	0.359978	-6.933057
161	6	0	-4.785867	1.749045	-6.879837
162	7	0	-5.038286	0.529111	5.035606
163	8	0	-4.224949	0.106393	2.953084
164	6	0	-5.212869	1.927402	4.962617
165	6	0	-5.778878	2.552926	6.091668
166	6	0	-6.046379	3.919872	6.080748
167	6	0	-5.762351	4.679718	4.946308
168	6	0	-5.185380	4.076945	3.814157
169	6	0	-4.900996	2.703545	3.839630
170	6	0	-4.644617	2.514563	-5.718923
171	6	0	-4.940845	3.886569	-5.719088
172	6	0	-5.362835	4.493005	-6.916127
173	6	0	-5.481925	3.740512	-8.085115
174	6	0	-5.199330	2.375430	-8.071186
175	7	0	-5.016992	4.900002	2.675402
176	7	0	-4.904786	4.717754	-4.571196
177	8	0	-4.307171	-8.558396	-3.317307
178	8	0	-4.682305	-8.506156	1.557532
179	6	0	-4.665589	-9.930775	-3.388572
180	6	0	-5.049213	-9.876931	1.602712
181	8	0	-4.087653	-6.148009	-6.108227
182	8	0	-4.729161	-1.967780	-8.459172
183	8	0	-4.883530	-6.004527	4.334180
184	8	0	-5.519673	-1.741179	6.528184
185	8	0	-6.050432	1.728893	7.158702
186	8	0	-6.031210	6.020271	4.830604
187	6	0	-6.650224	6.682062	5.922586
188	6	0	-6.655269	2.295978	8.310029
189	8	0	-5.645345	5.832974	-6.830868
190	8	0	-5.300890	1.559823	-9.170879
191	6	0	-5.725670	2.129240	-10.400312
192	6	0	-6.093280	6.506375	-7.997781

193	6	0	-4.431009	4.564297	1.491420
194	6	0	-4.742700	5.438043	0.295489
195	6	0	-4.425846	4.864458	-0.936594
196	6	0	-4.776538	5.366339	-2.182641
197	6	0	-4.472087	4.403350	-3.311024
198	6	0	-5.423597	6.674828	0.251863
199	6	0	-5.443112	6.616168	-2.189428
200	6	0	-5.742642	7.261129	-0.980251
201	8	0	-3.761005	3.539126	1.357936
202	8	0	-3.907139	3.345595	-3.034202
203	8	0	-5.781672	7.159116	-3.392603
204	8	0	-5.743455	7.258969	1.442789
205	6	0	-6.482456	8.400402	-3.447351
206	6	0	-6.456702	8.493898	1.464302
207	6	0	-5.383976	-6.698835	5.473469
208	6	0	-6.029292	-2.374478	7.697395
209	6	0	-5.028520	-2.648506	-9.676109
210	6	0	-4.387365	-6.904263	-7.279539
211	1	0	-3.557586	-4.890492	-0.851481
212	1	0	-4.725811	-9.681835	-0.885605
213	1	0	-4.480549	-6.460776	2.384787
214	1	0	-4.016498	-6.533101	-4.149841
215	1	0	-3.647316	-2.154275	-4.105643
216	1	0	-4.601160	-4.617859	-8.240679
217	1	0	-5.543056	-4.400639	6.347778
218	1	0	-3.949052	-2.073564	2.335582
219	1	0	-4.702173	-0.073291	-7.837126
220	1	0	-5.345047	0.100426	5.900030
221	1	0	-6.490275	4.390186	6.947869
222	1	0	-4.457845	2.232410	2.979082
223	1	0	-4.317895	2.038781	-4.811657
224	1	0	-5.802874	4.214961	-9.002357
225	1	0	-5.478729	5.801061	2.717724
226	1	0	-5.302165	5.640540	-4.697946
227	1	0	-4.673541	-10.189432	-4.448891
228	1	0	-3.932542	-10.562018	-2.869874
229	1	0	-5.662750	-10.107563	-2.965091
230	1	0	-5.216278	-10.113265	2.655371
231	1	0	-5.972335	-10.064361	1.039227
232	1	0	-4.248794	-10.518647	1.212482
233	1	0	-6.775854	7.722447	5.616953
234	1	0	-7.633268	6.249170	6.149374
235	1	0	-6.021232	6.645837	6.821630
236	1	0	-6.788105	1.477673	9.020652
237	1	0	-6.015154	3.065176	8.762030
238	1	0	-7.634757	2.732192	8.075509
239	1	0	-5.737885	1.314232	-11.126433
240	1	0	-6.734578	2.553865	-10.318266
241	1	0	-5.030413	2.906155	-10.743502
242	1	0	-6.258175	7.545028	-7.705142
243	1	0	-5.338007	6.473541	-8.793508
244	1	0	-7.034310	6.081954	-8.371043
245	1	0	-3.887998	3.933769	-0.920751
246	1	0	-6.246949	8.217144	-0.999540
247	1	0	-6.642291	8.601054	-4.507102
248	1	0	-7.450276	8.332039	-2.936993
249	1	0	-5.886337	9.209219	-3.009291
250	1	0	-6.600454	8.732358	2.518665
251	1	0	-5.876343	9.291276	0.986202
252	1	0	-7.432262	8.398209	0.973542
253	1	0	-5.352598	-7.757081	5.211790
254	1	0	-4.753217	-6.521038	6.352628

255	1	0	-6.417307	-6.406361	5.693252
256	1	0	-6.270821	-1.566006	8.388340
257	1	0	-6.936522	-2.947848	7.474747
258	1	0	-5.277939	-3.031641	8.151826
259	1	0	-5.238688	-1.866551	-10.406398
260	1	0	-4.173710	-3.244449	-10.016364
261	1	0	-5.909516	-3.291337	-9.564029
262	1	0	-4.314006	-7.950857	-6.982012
263	1	0	-5.402028	-6.695311	-7.638445
264	1	0	-3.662077	-6.700295	-8.075515
265	6	0	4.696063	1.110103	-0.759433
266	7	0	5.473440	0.057640	-1.089729
267	6	0	4.987431	-1.195872	-1.185492
268	6	0	3.641268	-1.417309	-0.989754
269	6	0	2.773830	-0.345005	-0.726191
270	6	0	3.344856	0.933274	-0.583709
271	6	0	1.336746	-0.603489	-0.655312
272	6	0	-1.637712	-1.161116	-0.860609
273	6	0	-2.989921	-1.278633	-1.091975
274	7	0	-3.753198	-0.162658	-1.110847
275	6	0	-3.262489	1.070144	-0.865558
276	6	0	-1.919984	1.221482	-0.613207
277	6	0	0.376983	0.331697	-0.510637
278	6	0	-1.062862	0.104983	-0.642810
279	1	0	5.165127	2.064298	-0.580494
280	1	0	6.456137	0.219551	-1.321397
281	1	0	5.679482	-2.006139	-1.394858
282	1	0	3.277672	-2.436288	-1.055823
283	1	0	2.762422	1.802014	-0.303030
284	1	0	1.058828	-1.643368	-0.810019
285	1	0	-1.053858	-2.072326	-0.868122
286	1	0	-3.476852	-2.226320	-1.266933
287	1	0	-4.739969	-0.255773	-1.331335
288	1	0	-3.951621	1.898251	-0.900140
289	1	0	-1.548717	2.219415	-0.412274
290	1	0	0.656729	1.371416	-0.358078

Standard orientation for the optimized structure of **1a ⊃ G4•2H**

Center Number	Atomic Number	Atomic Type	Coordinates (Angstroms) X	Y	Z
1	6	0	-10.018287	-1.375378	-1.270832
2	6	0	-8.563766	-1.355244	-1.729202
3	6	0	-7.852384	-0.023253	-1.954290
4	6	0	-8.597513	1.287542	-1.720541
5	6	0	-10.052034	1.267408	-1.262171
6	6	0	-10.762375	-0.063453	-1.037384
7	7	0	-7.820640	-2.663975	-1.962335
8	7	0	-7.888163	2.616375	-1.945034

9	6	0	-6.368248	-2.643922	-2.419938
10	8	0	-5.658842	-1.315208	-2.644483
11	6	0	-5.625123	-3.952654	-2.653071
12	6	0	-6.435685	2.636434	-2.402750
13	8	0	-5.692644	1.327653	-2.635861
14	6	0	-5.726281	3.965192	-2.627204
15	6	0	-4.270864	3.985289	-3.085869
16	6	0	-3.560440	5.316200	-3.310678
17	6	0	-4.304611	6.628075	-3.077208
18	6	0	-5.760080	6.608009	-2.618672
19	6	0	-6.470430	5.276000	-2.393772
20	6	0	-6.335493	-5.283639	-2.428224
21	6	0	-5.591344	-6.594447	-2.661657
22	6	0	-4.135843	-6.574301	-3.120343
23	6	0	-3.425503	-5.243439	-3.345131
24	6	0	-4.169567	-3.932582	-3.111720
25	6	0	-1.973025	-5.223381	-3.802846
26	6	0	-2.107962	5.336258	-3.768394
27	8	0	-1.364920	4.027478	-4.001505
28	7	0	-1.398610	6.665048	-3.992976
29	6	0	0.053866	6.685150	-4.450602
30	7	0	-1.229899	-6.532112	-4.035979
31	8	0	-1.263675	-3.894548	-4.027339
32	6	0	0.222577	-6.512010	-4.493605
33	6	0	0.966664	-7.823935	-4.727053
34	6	0	2.422166	-7.803788	-5.185739
35	6	0	3.132569	-6.471854	-5.410601
36	6	0	2.388418	-5.161002	-5.177079
37	6	0	0.932865	-5.181118	-4.718520
38	6	0	0.797931	5.374292	-4.684013
39	6	0	2.253486	5.394364	-5.142661
40	6	0	2.963856	6.725350	-5.367508
41	6	0	2.219685	8.037225	-5.134039
42	6	0	0.764186	8.017034	-4.675442

43	7	0	3.097770	-3.832212	-5.401661
44	7	0	2.996612	4.085633	-5.375794
45	8	0	-10.795076	2.576189	-1.029060
46	8	0	-10.727639	-2.704167	-1.046249
47	6	0	-12.247552	2.556087	-0.571435
48	6	0	-12.180031	-2.724220	-0.588645
49	8	0	-7.922906	5.255898	-1.936146
50	8	0	-3.595259	7.956864	-3.301791
51	8	0	-7.787887	-5.303648	-1.970531
52	8	0	-3.392778	-7.884148	-3.353491
53	8	0	0.257260	-9.152693	-4.502598
54	8	0	4.584961	-6.451801	-5.868205
55	6	0	5.328087	-7.760532	-6.101338
56	6	0	1.000386	-10.461424	-4.735731
57	8	0	4.416250	6.745359	-5.825202
58	8	0	0.021144	9.325815	-4.442331
59	6	0	0.730494	10.654648	-4.666824
60	6	0	5.125740	8.074123	-6.049769
61	6	0	2.357809	-2.527648	-5.169428
62	6	0	3.064226	-1.203170	-5.393098
63	6	0	2.324149	0.100354	-5.160918
64	6	0	3.030564	1.424875	-5.384497
65	6	0	2.290196	2.761112	-5.152215
66	6	0	4.511581	-1.183157	-5.849136
67	6	0	4.478128	1.437432	-5.840576
68	6	0	5.212076	0.128585	-6.070873
69	8	0	0.910315	-2.547635	-4.713406
70	8	0	0.842610	2.749621	-4.696099
71	8	0	5.191629	2.757795	-6.066410
72	8	0	5.252659	-2.487691	-6.081579
73	6	0	6.644198	2.769401	-6.524129
74	6	0	6.705137	-2.467633	-6.539295
75	6	0	-8.497291	-6.632406	-1.746076
76	6	0	-4.102131	-9.212938	-3.128909

77	6	0	-4.338301	9.265645	-3.068680
78	6	0	-8.665948	6.564678	-1.703035
79	1	0	-6.803409	-0.008580	-2.284837
80	1	0	-11.811432	-0.077764	-0.706793
81	1	0	-8.292005	-3.547070	-1.813104
82	1	0	-8.381954	3.485980	-1.790173
83	1	0	-3.734099	3.039971	-3.254255
84	1	0	-6.296959	7.553153	-2.450204
85	1	0	-6.103719	-7.554170	-2.499521
86	1	0	-3.657247	-2.972784	-3.273894
87	1	0	-1.892402	7.534696	-3.838025
88	1	0	-1.701234	-7.415082	-3.886809
89	1	0	2.959099	-8.749007	-5.354169
90	1	0	0.396155	-4.235874	-4.550096
91	1	0	0.285610	4.414495	-4.521838
92	1	0	2.732200	8.996878	-5.296249
93	1	0	4.062924	-3.819134	-5.705775
94	1	0	3.961713	4.099198	-5.679924
95	1	0	-12.615322	3.600238	-0.456341
96	1	0	-12.867474	2.026412	-1.329052
97	1	0	-12.321413	2.027215	0.405088
98	1	0	-12.521064	-3.778161	-0.480413
99	1	0	-12.266868	-2.204259	0.391644
100	1	0	-12.813546	-2.205121	-1.342260
101	1	0	6.369202	-7.543281	-6.429588
102	1	0	5.352534	-8.346236	-5.155297
103	1	0	4.805879	-8.348165	-6.889238
104	1	0	0.321845	-11.317220	-4.521282
105	1	0	1.337513	-10.512387	-5.795174
106	1	0	1.884501	-10.511084	-4.061367
107	1	0	0.030358	11.491414	-4.446844
108	1	0	1.612606	10.722335	-3.991510
109	1	0	1.066850	10.721193	-5.725700
110	1	0	6.171967	7.885717	-6.379290

111	1	0	4.588727	8.652811	-6.834240
112	1	0	5.134851	8.654687	-5.100175
113	1	0	1.275090	0.085631	-4.830349
114	1	0	6.261172	0.140026	-6.401402
115	1	0	6.990704	3.821371	-6.634050
116	1	0	7.274724	2.248754	-5.769055
117	1	0	6.728686	2.246884	-7.503232
118	1	0	7.072822	-3.511834	-6.654367
119	1	0	6.778504	-1.939276	-7.516135
120	1	0	7.325161	-1.937391	-5.782284
121	1	0	-9.543602	-6.444050	-1.416533
122	1	0	-8.506864	-7.212494	-2.695912
123	1	0	-7.960270	-7.211682	-0.961987
124	1	0	-3.401994	-10.049703	-3.348888
125	1	0	-4.437686	-9.279350	-2.069856
126	1	0	-4.984704	-9.280778	-3.803604
127	1	0	-3.659760	10.121441	-3.283129
128	1	0	-5.221929	9.315215	-3.743656
129	1	0	-4.676201	9.316644	-2.009384
130	1	0	-9.707063	6.347427	-1.374785
131	1	0	-8.143895	7.151776	-0.914656
132	1	0	-8.690095	7.151042	-2.648722
133	6	0	-5.897665	-1.472455	6.526192
134	6	0	-4.443090	-1.452396	6.067859
135	6	0	-3.725866	-0.120530	5.861658
136	6	0	-4.465014	1.190116	6.114311
137	6	0	-5.919505	1.170106	6.572621
138	6	0	-6.635856	-0.160730	6.778565
139	7	0	-3.706031	-2.760983	5.815935
140	7	0	-3.749683	2.518799	5.908721
141	6	0	-2.253553	-2.740925	5.358219
142	8	0	-1.538220	-1.412285	5.152540
143	6	0	-1.516408	-4.049506	5.106183
144	6	0	-2.297205	2.538857	5.451006

145	8	0	-1.560060	1.230276	5.198969
146	6	0	-1.581872	3.867497	5.245327
147	6	0	-0.126373	3.887688	4.786730
148	6	0	0.589896	5.218431	4.580719
149	6	0	-0.148295	6.530156	4.833092
150	6	0	-1.603712	6.510058	5.291756
151	6	0	-2.320097	5.178274	5.497715
152	6	0	-2.232677	-5.380249	5.312195
153	6	0	-1.494591	-6.691000	5.059790
154	6	0	-0.039038	-6.670885	4.601232
155	6	0	0.677315	-5.340092	4.395198
156	6	0	-0.060855	-4.029391	4.647624
157	6	0	2.129707	-5.320040	3.937595
158	6	0	2.042426	5.238457	4.123131
159	8	0	2.779571	3.929876	3.871094
160	7	0	2.757705	6.567172	3.917414
161	6	0	4.210236	6.587155	3.459736
162	7	0	2.866852	-6.628621	3.685558
163	8	0	2.845040	-3.991400	3.731915
164	6	0	4.319384	-6.608638	3.227880
165	6	0	5.057575	-7.920363	2.975507
166	6	0	6.512990	-7.900222	2.516933
167	6	0	7.229374	-6.568438	2.310974
168	6	0	6.491150	-5.257661	2.563362
169	6	0	5.035651	-5.277852	3.021959
170	6	0	4.948405	5.276497	3.207400
171	6	0	6.403822	5.296595	2.748735
172	6	0	7.120227	6.627356	2.542829
173	6	0	6.381982	7.939156	2.795165
174	6	0	4.926481	7.919009	3.253851
175	7	0	7.206483	-3.929021	2.357683
176	7	0	7.141019	3.987982	2.496827
177	8	0	-6.656650	2.478688	6.824657
178	8	0	-6.612998	-2.801094	6.731871

179	6	0	-8.109126	2.458586	7.282283
180	6	0	-8.065474	-2.821196	7.189496
181	8	0	-3.772572	5.158172	5.955341
182	8	0	0.567036	7.858839	4.627502
183	8	0	-3.685153	-5.400351	5.769820
184	8	0	0.698130	-7.980533	4.349159
185	8	0	4.342242	-9.249003	3.181186
186	8	0	8.681850	-6.548336	1.853348
187	6	0	9.418995	-7.856917	1.601312
188	6	0	5.079387	-10.557584	2.929150
189	8	0	8.572618	6.647408	2.085226
190	8	0	4.189336	9.227590	3.505887
191	6	0	4.904614	10.556305	3.300170
192	6	0	9.287951	7.976048	1.879547
193	6	0	6.472452	-2.624618	2.608602
194	6	0	7.184712	-1.300265	2.403820
195	6	0	6.450702	0.003114	2.654791
196	6	0	7.162962	1.327468	2.450009
197	6	0	6.428622	2.663611	2.701503
198	6	0	8.632205	-1.280278	1.947797
199	6	0	8.610474	1.340056	1.993803
200	6	0	9.338578	0.031378	1.744709
201	8	0	5.025011	-2.644637	3.064752
202	8	0	4.981226	2.652063	3.157762
203	8	0	9.329904	2.660301	1.786744
204	8	0	9.367302	-2.584662	1.696450
205	6	0	10.782421	2.671938	1.328898
206	6	0	10.819694	-2.564609	1.238847
207	6	0	-4.400486	-6.728991	5.975500
208	6	0	-0.017203	-9.309173	4.554838
209	6	0	-0.170023	9.167426	4.879427
210	6	0	-4.509633	6.466803	6.207355
211	1	0	-2.676806	-0.105807	5.531090
212	1	0	-7.684830	-0.174991	7.109133

213	1	0	-4.181271	-3.643912	5.952534
214	1	0	-4.239484	3.388412	6.076133
215	1	0	0.406046	2.942450	4.604630
216	1	0	-2.136297	7.455153	5.473810
217	1	0	-2.011259	-7.650673	5.208341
218	1	0	0.455814	-3.069717	4.499074
219	1	0	2.267957	7.436710	4.084863
220	1	0	2.391610	-7.511506	3.822247
221	1	0	7.045575	-8.845317	2.334879
222	1	0	4.503234	-4.332657	3.203969
223	1	0	4.431736	4.316824	3.355950
224	1	0	6.898845	8.898684	2.646578
225	1	0	8.171637	-3.915943	2.053569
226	1	0	8.106120	4.001547	2.192697
227	1	0	-8.472114	3.502705	7.412412
228	1	0	-8.731520	1.942626	6.517380
229	1	0	-8.185216	1.916168	8.251212
230	1	0	-8.411068	-3.875038	7.282777
231	1	0	-8.149702	-2.314933	8.177176
232	1	0	-8.696678	-2.288459	6.443542
233	1	0	10.460918	-7.639732	1.276005
234	1	0	9.440930	-8.456266	2.538911
235	1	0	8.894032	-8.430933	0.805278
236	1	0	4.397038	-11.413270	3.131553
237	1	0	5.416029	-10.594903	1.868955
238	1	0	5.963312	-10.620892	3.602583
239	1	0	4.208286	11.392961	3.532196
240	1	0	5.787158	10.610422	3.976198
241	1	0	5.241076	10.636449	2.242248
242	1	0	10.333371	7.787708	1.547082
243	1	0	8.753452	8.568337	1.103427
244	1	0	9.299902	8.543045	2.837252
245	1	0	5.401643	-0.011608	2.985360
246	1	0	10.387675	0.042775	1.414090

247	1	0	11.133627	3.723782	1.233945
248	1	0	11.410774	2.137626	2.076327
249	1	0	10.864439	2.163093	0.342420
250	1	0	11.182766	-3.608679	1.108695
251	1	0	10.895290	-2.022707	0.269600
252	1	0	11.442327	-2.048065	2.003248
253	1	0	-5.445821	-6.540601	6.307942
254	1	0	-4.412785	-7.295337	5.017469
255	1	0	-3.865893	-7.321861	6.751125
256	1	0	0.679128	-10.145872	4.322722
257	1	0	-0.352894	-9.389308	5.612997
258	1	0	-0.900208	-9.363443	3.879429
259	1	0	0.512240	10.023106	4.677136
260	1	0	-1.053547	9.230638	4.205494
261	1	0	-0.507491	9.204855	5.939437
262	1	0	-5.551640	6.249568	6.532684
263	1	0	-3.984854	7.040159	7.003927
264	1	0	-4.531213	7.066692	5.270058
265	6	0	-3.005427	-1.285342	-5.671801
266	7	0	-2.884924	-0.071867	-6.584345
267	6	0	-2.094172	1.147632	-6.129189
268	6	0	-1.423974	1.153686	-4.761617
269	6	0	-1.544531	-0.059714	-3.849110
270	6	0	-2.335231	-1.279244	-4.304138
271	6	0	-0.874335	-0.053616	-2.481448
272	6	0	-0.083635	1.165914	-2.026420
273	6	0	0.586560	1.172012	-0.658757
274	6	0	0.466090	-0.041382	0.253637
275	6	0	-0.324610	-1.260912	-0.201391
276	6	0	-0.994806	-1.267010	-1.569054
277	6	0	1.136285	-0.035284	1.621299
278	6	0	1.926986	1.184246	2.076328
279	6	0	2.597181	1.190344	3.443990
280	7	0	2.476678	-0.023131	4.356534

281	6	0	1.685926	-1.242630	3.901378
282	6	0	1.015729	-1.248684	2.533806
283	1	0	-3.576454	-2.166114	-6.000481
284	1	0	-3.344719	-0.076098	-7.522715
285	1	0	-2.007187	2.024056	-6.788237
286	1	0	-0.852949	2.034502	-4.432847
287	1	0	-2.422270	-2.155593	-3.645128
288	1	0	0.003404	2.042263	-2.685430
289	1	0	1.157672	2.052833	-0.330099
290	1	0	-0.411649	-2.137261	0.457619
291	1	0	-1.565918	-2.147831	-1.897712
292	1	0	2.014024	2.060595	1.417317
293	1	0	3.168209	2.071116	3.772670
294	1	0	2.936475	-0.018944	5.294814
295	1	0	1.598942	-2.119054	4.560426
296	1	0	0.444703	-2.129500	2.205036

References

[1] Li, X.; Yuan, X.; Deng, P.; Chen, L.; Ren, Y.; Wang, C.; Wu, L.; Feng, W.; Gong, B.; Yuan, L. Macrocyclic Shape-Persistency of Cyclo[6]aramide Results in Enhanced Multipoint Recognition for the Highly Efficient Template-Directed Synthesis of Rotaxanes. *Chem. Sci.* **2017**, *8*, 2091–2100.

[2] Kothapalli, S.; Kannekanti, V.; Ye, Z.; Yang, Z.; Chen, L.; Cai, Y.; Zhu, B.; Feng, W.; Yuan, L. Light-Controlled Switchable Complexation by a Non-Photoresponsive Hydrogen-Bonded Amide Macrocycle. *Org. Chem. Front.* **2020**, *7*, 846–855.

[3] Li, X.; Li, B.; Chen, L.; Hu, J.; Wen, C.; Zheng, Q.; Wu, L.; Zeng, H.; Gong, B.; Yuan, L. Liquid-Crystalline Mesogens Based on Cyclo[6]aramides: Distinctive Phase Transitions in Response to Macrocyclic Host-Guest Interactions. *Angew. Chem. Int. Ed.* **2015**, *54*, 11147–11152.

[4] Han, Y.; Meng, Z.; Chen, C.-F., Acid/Base Controllable Complexation of a Triptycene-Derived Macrotricyclic Host and Protonated 4,4′-Bipyridinium/Pyridinium Salts. *Chem. Commun.* **2016**, *52*, 590-593.

[5] Gaussian 09, Revision E.01, M. J. Frisch, G. W. Trucks, H. B. Schlegel, G. E. Scuseria, M. A. Robb, J. R. Cheeseman, G. Scalmani, V. Barone, B. Mennucci, G. A. Petersson, H. Nakatsuji, M. Caricato, X. Li, H. P. Hratchian, A. F. Izmaylov, J. Bloino, G. Zheng, J. L. Sonnenberg, M. Hada, M. Ehara, K. Toyota, R. Fukuda, J. Hasegawa, M. Ishida, T. Nakajima, Y. Honda, O. Kitao, H. Nakai, T. Vreven, J. A. Montgomery, Jr., J. E. Peralta, F. Ogliaro, M. Bearpark, J. J. Heyd, E. Brothers, K. N. Kudin, V. N. Staroverov, T. Keith, R. Kobayashi, J. Normand, K. Raghavachari, A. Rendell, J. C. Burant, S. S. Iyengar, J. Tomasi, M. Cossi, N. Rega, J. M. Millam, M. Klene, J. E. Knox, J. B. Cross, V. Bakken, C. Adamo, J. Jaramillo, R. Gomperts, R. E. Stratmann,O. Yazyev, A. J. Austin, R. Cammi, C. Pomelli, J. W. Ochterski, R. L. Martin, K. Morokuma, V. G. Zakrzewski, G. A. Voth, P. Salvador, J. J. annenberg, S. Dapprich, A. D. Daniels, O. Farkas, J. B. Foresman, J. V. Ortiz, J. Cioslowski, and D. J. Fox, Gaussian, Inc., Wallingford CT, **2013**.
